# A Supramolecular Nanosheet Assembled from Carpyridines and Water

**DOI:** 10.1021/jacs.4c17024

**Published:** 2025-05-19

**Authors:** Joseph F. Woods, Kai Zhang, Joëlle Peterschmitt, Olivier Blacque, Céline Besnard, Gustavo Santiso-Quinones, Laura Samperisi, Andreas Vargas Jentzsch, Michel Rickhaus

**Affiliations:** † Department of Chemistry, 27217 University of Zurich, 8057, Zurich, Switzerland; ‡ Department of Organic Chemistry, 27212 University of Geneva, 1205, Geneva, Switzerland; § Laboratoire de Cristallographie, 27212 University of Geneva, 1211, Geneva, Switzerland; ∥ ELDICO Scientific AG, 4123, Allschwil, Switzerland; ⊥ SAMS Research Group, University of Strasbourg, Institut Charles Sadron, CNRS, 67200, Strasbourg, France

## Abstract

The use of water as a solvent to facilitate supramolecular self-assembly and polymerization is well-documented; however, it is rare that water acts as a monomer that undergoes polymerization. We report the formation of nanosheets composed of water and a saddle-shaped porphyrinoid macrocycle, carpyridine, which allows for linearly stacked, eclipsed columns within formed 2D structures. Self-assembling carpyridine monomers from solutions with different extents of wetness permit the formation of nanosheets that appear identical by microscopy. Structural analysis through electron diffraction reveals fundamental changes in the local organization. Under dry conditions, carpyridine stacks are formed through π–π interactions between curved surfaces, whereas in solutions containing greater quantities of water, a hydrogen-bonded water-to-carpyridine-core network is propagated throughout perfectly linear columns. The observed wet phase can be interconverted to a dry one through vapor annealing, indicating an accessible energy surface of polymorphism.

## Graphical abstract

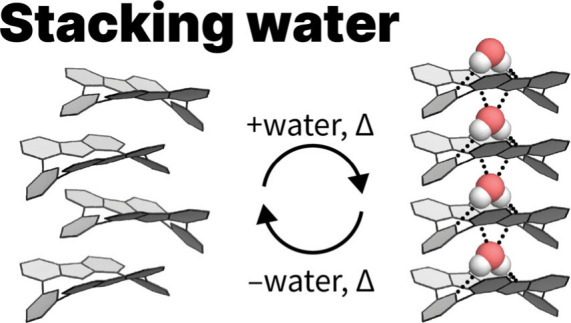

The impact of water upon the capability of a monomer to self-assemble is well-known and carefully considered in supramolecular chemistry.^1–3^ Self-assembly can be promoted or prevented by the presence of water, or even prompt a change of assembly mode upon its addition.^4–7^ When employed as a solvent or a cosolvent for polymerization, water often exploits the hydrophobic character of monomers to organize matter in a manner that excludes the aqueous media or affects the strength of a hydrogen bond.^8–12^ An alternative manner to facilitate or disrupt assembly is through direct coordination of the water molecules to specific sites on the assembling monomer, such as a group capable of forming hydrogen bonds.^13–15^

The affinity of water to bind to the monomer varies in each case; however, it is important to acknowledge that even in low quantities, water may have a drastic effect upon the polymerization.^16,17^ This is particularly notable in systems where multiple, weaker interactions are at play.^18,19^ The role of water is often speculated upon or implied in supramolecular polymerization as direct evidence of its precise involvement is difficult to obtain.^20^ Herein, we present, to the best of our knowledge, the first known case of a supramolecular material that incorporates water as a monomer, which is supported by crystallographic evidence.

Carpyridines are porphyrinoids that our group has used to exert control over self-assembly processes by leveraging molecular curvature to govern weak interactions, resulting in the formation of columnar assemblies within 2D nanosheets and 1D fibers.^6,21–23^ Incorporation of negative curvature within the molecule limits the extent of rotational and translational freedom individual units have when aggregated into linear columns (Figure 1, left).^6,22^ These systems are weakly organized, which permits modulation of the crystallinity from crystal into a soft system by simply trimming or expanding the side chains by one or two carbons. The heteroatom-rich core is not engaged in binding and is not a driver for the assembly. We rationalized that this vacancy could act as a preorganized framework to host water at the expense of π–π interactions (Figure 1, right). Tuning the crystallinity well enough should consequently allow for probing the organization of the units within the self-assembled materials.

**Figure 1:**
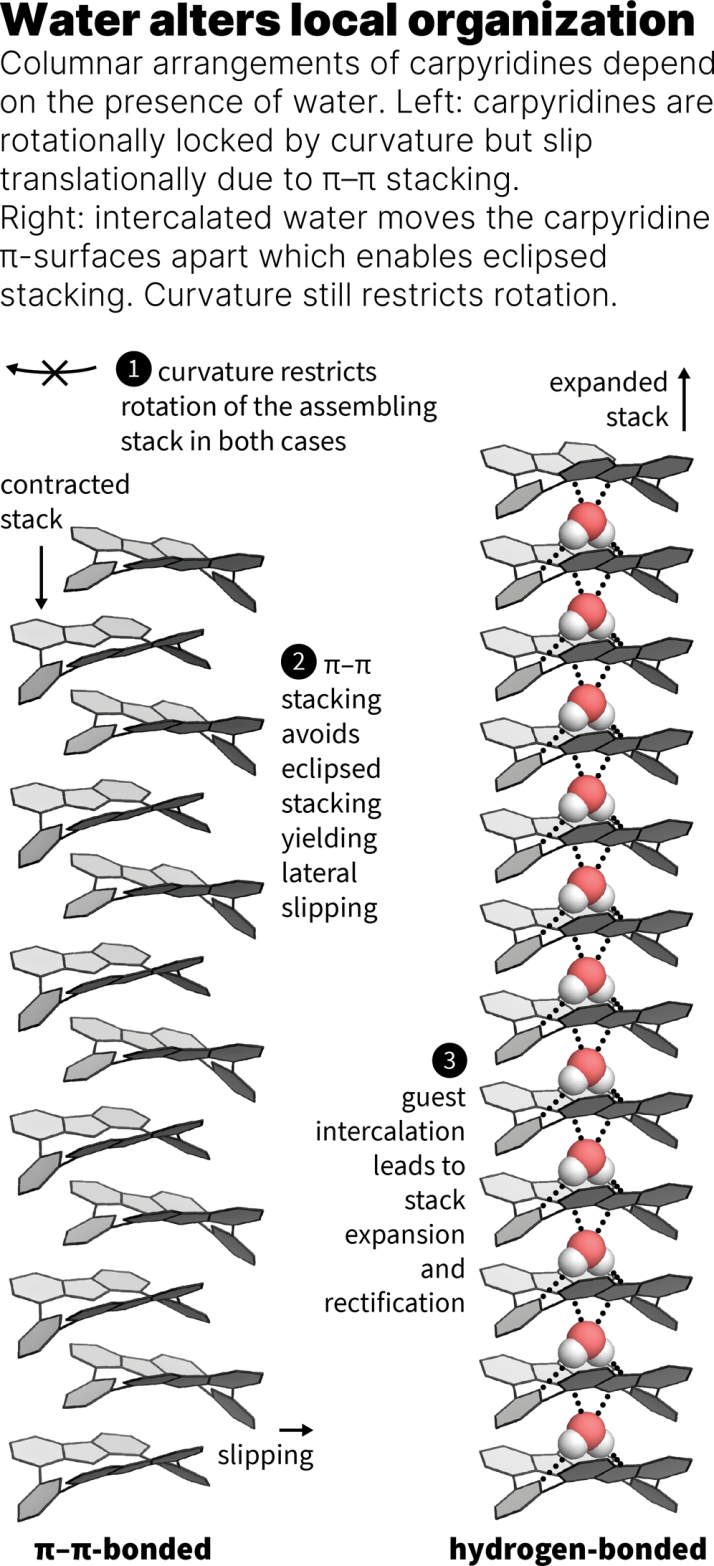
Different columnar arrangements of carpyridines are possible depending on the presence of water. Left: crystal structure of **2H-Car-Ph** grown from toluene/methanol, right: μ-ED (r.t.) of **2H-Car-Ph** nanosheets grown from wet toluene.

We sought to prepare a more rigidified system through restriction of the disorder typically associated with alkyl side chains at the periphery of the macrocycle. Retaining a six-carbon count in the side chain was ideal for assembly but cyclization and planarization into an aryl ring (phenyl) would remove significant disorder. We rationalized that the aromatic side chain would likely favor a coplanar arrangement with the carbazole and strengthen the shape-governing effect.^21,22^ In addition, the phenyl rings provide an opportunity for further π–π interactions that rigidify the self-assembled structure. This should make such a system suitable for diffractive analyses, such as microelectron diffraction (μ-ED), to identify the assembly packing mode.

Synthesis began through functionalization at the 3- and 6-positions of the carbazole with a phenyl ring to prepare a more electron rich system (Scheme 1). Phenylboronic acid was reacted with 3,6-dibromocarbazole via a Suzuki cross-coupling with Pd­(PPh_3_)_4_ to provide **1**. This was then subjected to bromination and subsequent Miyaura borylation conditions. Suzuki-Miyaura cross-coupling of one equivalent of **3** with an initial half an equivalent of dibromopyridine, followed by sequential addition of a further half equivalent yielded the desired phenylated carpyridine, **2H-Car-Ph**, in an improved yield of 21% compared to alkyl derivatives.^6,21–23^ UV–vis and fluorescence emission spectroscopy in toluene (Figure S7) returned the expected optical profiles of a carpyridine but also showed a red-shift in maxima compared to the previous alkyl derivatives.^6,21–23^ The shift of the maxima to lower energies of 319 and 380 nm in the absorption spectrum indicated coupling between the carbazole and benzene ring systems. 2H-Car-Ph exhibited a moderately strong fluorescence quantum yield of 50% from a single band at 397 nm.

**Scheme 1:**
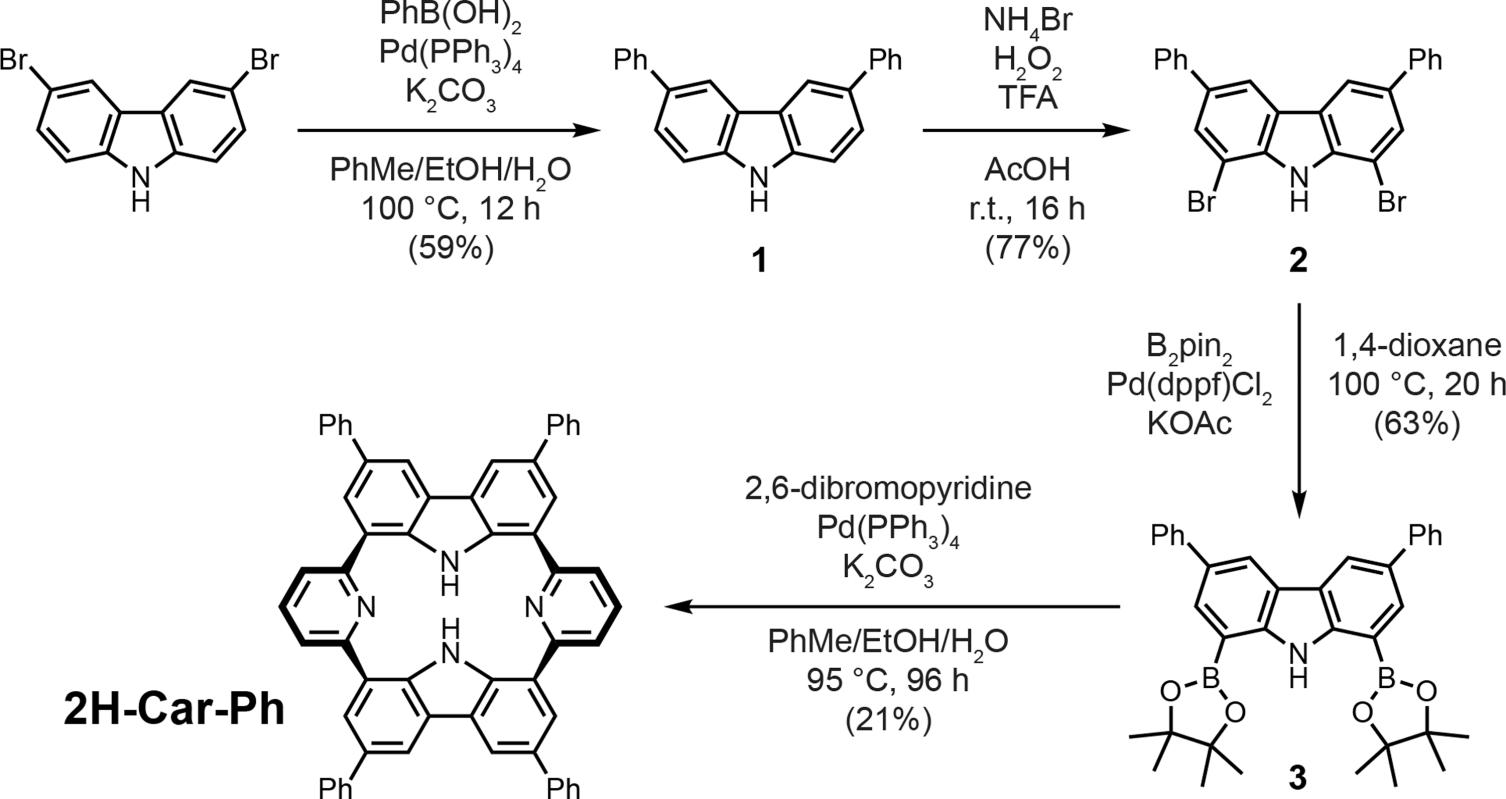
Synthetic Route towards **2H-Car-Ph** ^a^

a The macrocyclization reaction follows an optimized procedure involving sequential addition of the 2,6-dibromopyridine to favor carpyridine formation.

The carpyridine was dissolved in dry toluene (13 ppm, determined via Karl Fischer titration) to provide a 1 mM solution as water was perceived to hinder assembly formation.^6^ After heating and cooling back to room temperature, the solution was dropcasted onto a C/Cu transmission electron microscopy (TEM) grid to determine the self-assembly capability on-surface. Examination of the sample under the TEM beam showed the presence of multimicrometer long nanosheets that were notably thinner than those seen with the alkyl derivatives (Figure 2).^21,22^

**Figure 2:**
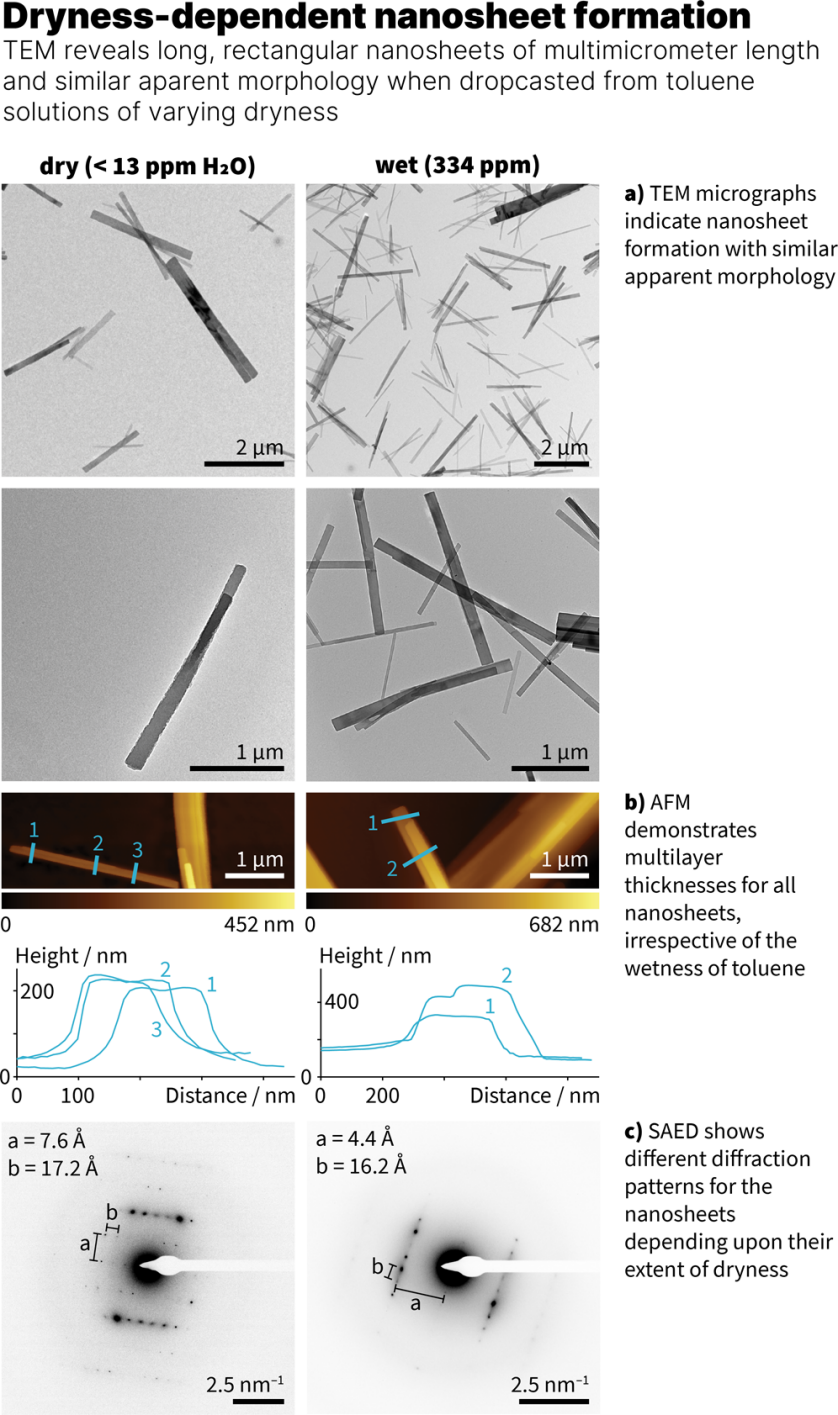
TEM micrographs, AFM images and height traces, and SAED patterns of **2H-Car-Ph** nanosheets from dry and wet toluene.

To assess whether the nanosheets were formed using regular toluene with a higher water content (101 ppm) due to storage under ambient conditions, further 1 mM solutions were prepared, dropcasted and visualized under the TEM (Figure S14). This revealed similar rectangular objects on surface with arguably smoother nanosheet edges and prompted questioning of whether assembly formation was possible in a solvent that was deliberately wetted. A solution of the same concentration of **2H-Car-Ph** in wet toluene (334 ppm) was then prepared, which again provided nanosheets of similar thicknesses, lengths and well-defined edges (Figure 2). For all three conditions used to prepare **2H-Car-Ph** nanosheets, atomic force microscopy (AFM) was used to confirm the uniformity and height of the assemblies, which is in the range of tens to hundreds of nanometers, highlighting their multilayered nature (Figure 2 and Figure S19).

Insights into the molecular structure within the nanosheets were provided from the selected area electron diffraction (SAED) patterns in TEM (Figure 2, Figure S16). Discrete diffraction spots and their spacings in two dimensions demonstrated that different compositions were observed depending upon the water content of the toluene within the **2H-Car-Ph** solutions. The SAED patterns with dry toluene provided different values (7.6 Å × 17.2 Å) to those seen with regular and wet toluene (4.2 Å × 16.4 Å and 4.4 Å × 16.2 Å, respectively), suggesting that a different assembly is obtained when using dry toluene instead of regular or wet toluene. The stability of the nanosheets under the electron beam formed with dry toluene was noticeably greater than those prepared from regular or wet toluene.

Single-crystals of **2H-Car-Ph** were grown from vapor diffusion of methanol into regular toluene to examine which structure is adopted in the macroscopic crystalline state and to deduce the degree of coplanarization within the molecular saddle. Lattice parameters were in agreement with the 2D parameters obtained from the SAED pattern with dry toluene and the carbazole and phenyl rings shared a similar plane, indicating a larger saddle topography. Propagating the crystal structure packing revealed an environment of antiparallel columnar arrays containing **2H-Car-Ph** molecules (Figure 3) with a slight lateral translational offset. The addition of the phenyl ring appeared to impose a heavy restriction upon both rotational and translational motions, more so than the rotational locking effect seen with alkyl carpyridines.^22^ The maximum value of permitted rotation is lowered to 0.45° between carpyridines and, crucially, significant contractions in the columnar width were realized such that **2H-Car-Ph** is only found in two distinct environments. Individual columns are then further rigidified through lateral CH−π interactions with neighboring columns, and the presence of an additional toluene solvent molecule within the unit cell assists the expansion of the structure into three dimensions.

**Figure 3:**
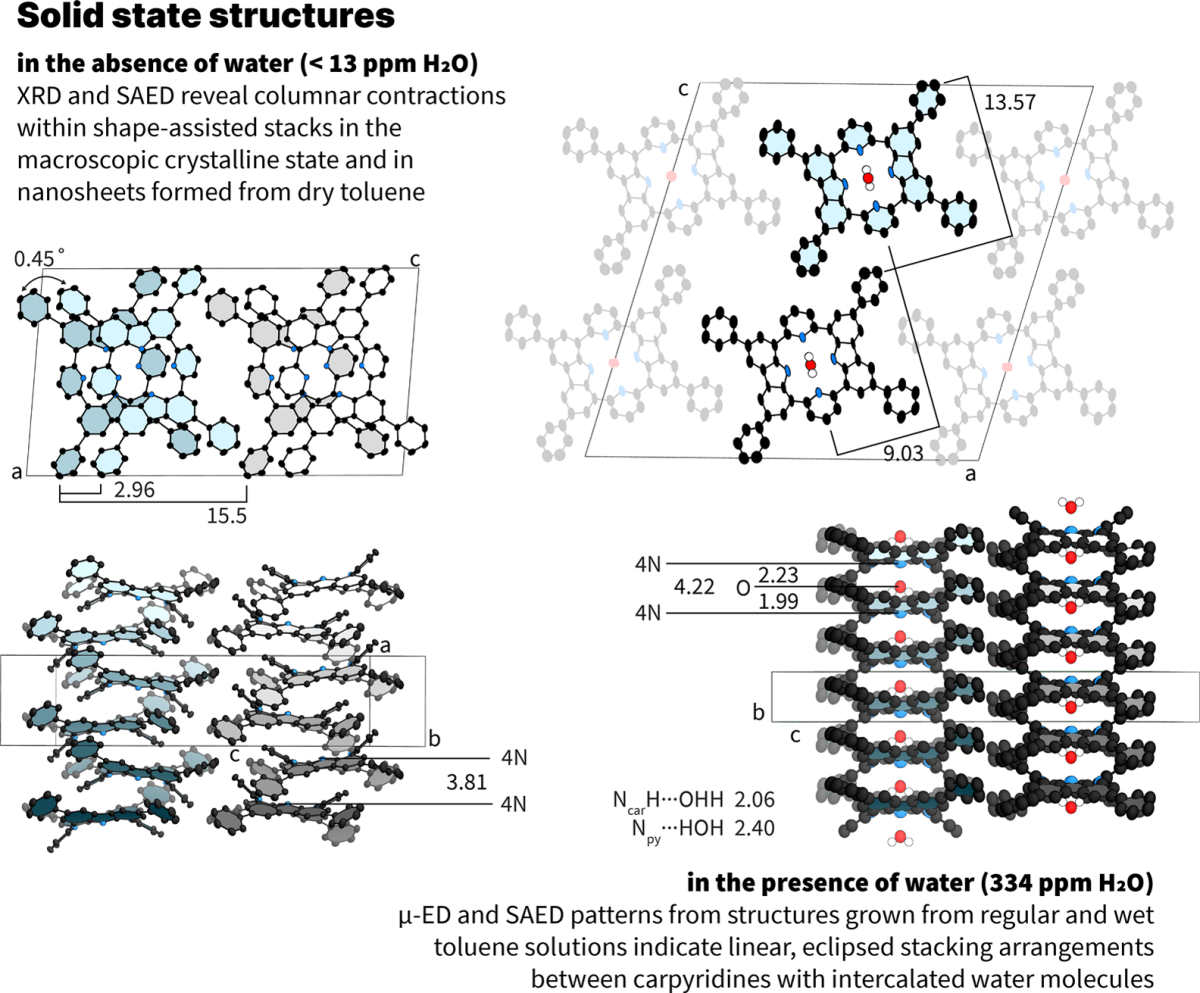
Structure of **2H-Car-Ph** nanosheets in dry conditions, left, and in the presence of water, right. Hydrogen atoms are omitted for clarity.

Determining the structure of the nanosheets formed from wet toluene relied upon using μ-ED (r.t.) with an ELDICO ED-1 diffractometer due to the sensitivity of the sample to the electron beam with other techniques. Carpyridines were observed in perfectly eclipsed antiparallel columns, reinforced through a hydrogen-bonding interaction with a coassembling water molecule inserted in the central cavity of the macrocycle (Figure 3). The water molecule forces the carpyridine macrocycles to be 4.22 Å apart from one another (vs 3.81 Å observed in dry samples) with connecting hydrogen bonds from the carbazole N–H groups to the oxygen of water (2.06 Å) and from the hydrogens of water to the pyridines of **2H-Car-Ph** (2.40 Å). This second hydrogen bond appears to be weaker due to the longer bond length. A single water molecule between two carpyridines holds the structure together, overcoming the π–π interactions responsible for the assembly in dry toluene. This interaction nullifies any rotational or translational offsets and also alters the packing of the carpyridines such that voids form between columnar arrays (Figure S33). These voids are propagated throughout the structure, leading to porous 1D channels within the nanosheets that account for 14% volume of the unit cell.

When examining samples of **2H-Car-Ph** from regular toluene at low temperatures, cryo-ED (100 K) revealed a structure that appears to exist in a form between dry and wet conditions. Carpyridines were seen to stack into slightly slipped antiparallel columns but with an intercalated water molecule between each macrocycle (Figure S31). The tripling of the unit cell along the stacking direction allows distinct carpyridine and water environments to exist in the column due to small translational offsets, yielding an average rotation of 5.6° between carpyridines. Voids that lead to porous channels throughout the nanosheets are found between carpyridine columns but are less well-defined compared to the structure obtained from μ-ED (r.t.). This reorganization of the non-dried sample hints toward the existence of other semistable intermediate states. Conversion of the wet phase to the dry phase through annealing in dry toluene succeeded as evidenced by powder X-ray diffraction (PXRD, Figures S34 and S35). The inverse transformation, annealing dry nanosheets in water-saturated toluene, did not result in a phase change, which we believe to be a consequence of the dry polymorph being more compact overall. Full dissolution into the monomeric state, removal of solvent and redissolving in wet toluene does then allow access to the other polymorph.

To assess whether association occurs in solution for **2H-Car-Ph**, variable temperature (VT) UV–vis and dynamic light scattering (DLS) experiments were performed in dry toluene (Figure S8). There was no spectroscopic indication of aggregation as only linear changes were detected when changing temperature. There were no apparent changes to the absorption signatures in relation to the dryness of the toluene solution (dry, regular or wet; Figure S9). However, VT DLS shows that solutions of **2H-Car-Ph** in dry toluene (600 μM) contain nanosheets that fully dissolve at 90 °C and reform upon cooling down to room temperature (Figure S37 – S41). This suggests that neither dry nor wet self-assembled polymorphs show sufficient orbital overlap to result in significant changes to the UV–vis spectra.

When titrating trifluoroacetic acid (TFA) into a toluene solution of **2H-Car-Ph**, a new band in the absorption spectrum at 430 nm evolved (Figure S10). However, no assemblies were visualized with TEM (Figure S17). The addition of TFA allowed for easier growth of single-crystals suitable for analysis, but the obtained carpyridine-TFA crystal structures are clearly distinct from the ones discussed earlier. Notably the coassembly by coordination to the TFA anion is clearly evidenced. This result indicates that the spectroscopic observations are to be attributed to the coordination complex formation (Figures S26 and S27) rather than an aggregated state.

While the structures described here correspond to self-assembled nanosheets and not to classical supramolecular polymers, the molecular insights of the extreme role that water can take are most intriguing. Indeed, although there are reports of water facilitating the supramolecular polymerization of monomers,^3,20,24,25^ the elucidation of the role of water has not been achieved to our knowledge. Given that other alkylated carpyridine systems can form 1D supramolecular polymers,^6^ we speculate and are currently working on the design of new carpyridines that will allow us to investigate this topic in detail.

**2H-Car-Ph** is arguably an ideal molecular system to study self-assembled materials with water because it is known that phenyl rings can assist with water incorporation.^26,27^ However, their observation was only made possible due to the appropriate use of the relatively new technique of electron diffraction crystallography. Classical techniques either failed to resolve the structure or gave visually identical appearances, underpinning how important a careful analysis is required to reveal the local organization within a self-assembled structure.

The uncovered porous channels within the water-containing nanosheets pose an opportunity for host–guest chemistry, like gas adsorption, as small molecules could become encapsulated within the porous material. The discovery also presents the opportunity to utilize and tune carpyridines to become ligands that influence supramolecular ordering. Single-crystal X-ray diffraction has also shown that π-extension of the carpyridine core increases the shape-assistance effect from a larger saddle to restrict the disorder in the formed columnar stacks, adding weight to the shape-assisted self-assembly argument. Further π-extension and intercalation studies are currently in progress with the goal of expanding and unifying our observations to linear supramolecular polymers in solution.

## Supplementary Material



## Notes

Additional experimental details, materials and methods, including NMR characterization, spectroscopic data, microscopy, DFT calculations, solid state characterization and analysis and dynamic light scattering (PDF)
