# Supplementary material for: A Supramolecular Nanosheet Assembled from Carpyridines and Water
Source: J Am Chem Soc. 2025 May 19;147(22):18380–5. doi: 10.1021/jacs.4c17024 (PMC12147159; doi:10.1021/jacs.4c17024)
Supplement: Supplementary file 1 [file ja4c17024_si_001.pdf]

Supplementary Information for:

A Supramolecular Nanosheet Assembled from Carpyridines And Water

Joseph F. Woods,[a]‡ Kai Zhang,[a,b]‡ Joëlle Peterschmitt,[c] Olivier Blacque,[a] Céline Besnard,[c] Gustavo Santiso-Quinones,[d] Laura Samperisi,[d] Andreas Vargas Jentzsch,[e] Michel Rickhaus*[a,b]

[a]Department of Chemistry, University of Zurich, 8057, Switzerland
[b]Department of Organic Chemistry, University of Geneva, 1205, Geneva, Switzerland
[c]Laboratoire de Cristallographie, University of Geneva, 1211, Geneva, Switzerland
[d]ELDICO Scientific AG, 4123, Allschwil, Switzerland
[e]SAMS Research Group, University of Strasbourg, Institut Charles Sadron, CNRS, 67200, Strasbourg, France

‡These authors contributed equally

***Corresponding author: michel.rickhaus@unige.ch**

Contents:

1. **Methods**
2. **Experimental Procedures**
3. **NMR Characterization**
4. **Spectroscopic Data**
5. **Microscopy**
6. **DFT Calculations**
7. **Solid State Characterization and Analysis**
8. **Dynamic Light Scattering**

Methods

General Information

All reagents and solvents were purchased from Avantor, Chemie Brunschwig AG, Sigma-Aldrich or Thermo Fisher and used without further purification. Dry solvents were obtained using a solvent purification system (Pure Solv PS-MD-4EN, Innovative Technology Inc.) equipped with alumina drying columns under argon. Reaction control was performed using analytical thin layer chromatography (TLC) on aluminium sheets coated with silica gel 60 F254 (Merck) or gas chromatography-mass spectrometry (GCMS) (Shimadzu Gas Chromatograph GC-2010 Plus, GCMS-QP2010 SE). Visualization of TLC plates was achieved using UV light at 254 or 366 nm. Flash column chromatography (FC) was performed using SiO_2 (60 Å, 230–400 mesh, particle size 0.063–0.200 mm). Preparative gel permeation chromatography (GPC) was carried out on a Shimadzu recycling GPC system equipped with a LC-20AR prominence liquid chromatograph pump, an SPDM40 photodiode array detector, a DGU-403 degassing and a CBM-40 system controller using two ReproGel 500 GPC columns (5 µm, 20×600 mm) with chloroform as the eluent passing through at a rate of 3.5 mL per minute.

All *NMR spectra* were recorded on AV2-400 MHz Bruker spectrometers at 298 K unless stated otherwise. Chemical shifts are given in ppm and the spectra are calibrated using the residual chloroform signals (7.26 ppm for ^{1}H NMR and 77.00 ppm for ^{13}C NMR). Coupling constants J are given in Hz and multiplicities are abbreviated as follows: s (singlet), d (doublet), t (triplet), m (multiplet), br (broad).

High resolution mass spectra (HR-MS) were recorded by the Mass Spectrometric Service at the University of Zurich on a Dionex Ultimate 3000 UHPLC system (ThermoFischer Scientifics, Germering, Germany) connected to a QExactive MS with a heated ESI source (ThermoFisher Scientific, Bremen, Germany) or on a double-focusing (BE geometry) magnetic sector mass spectrometer DFS (ThermoFisher Scientific, Bremen, Germany) with a heated EI source.

UV-vis absorbance measurements were recorded at 298 K or at the given temperature with a Shimadzu UV-Visible Spectrophotometer UV-1900 Series using quartz 1 cm or 1 mm cuvettes.

Fluorescence measurements were carried out using a calibrated Edinburgh Instruments FS5 spectrofluorometer equipped with an SC-25 Temperature Controlled Holder TE-Cooled-Standard cell for emission spectra and an SC-30 Integrating Sphere cell for obtaining quantum yields. All solvents used for spectrophotometric analysis were of analytical grade.

Infrared spectra were recorded in a Spectrum Two FT-IR spectrometer (Perkin–Elmer) equipped with an universal ATR.

Dynamic light scattering (DLS) measurements were performed using a Malvern type Zetasizer Nano ZSP.

Sample Preparation

Dry toluene was prepared by sparging with nitrogen and then storing over activated 3 Å molecular sieves overnight in a sealed vial with septum. The water content for dry toluene prepared with this method was 12.6 ppm determined by DL32 Coulometric Karl-Fischer Titrator. Dry samples were prepared by carefully transferring the dry toluene into the oven-dried sealed sample vial with septum under nitrogen atmosphere.

As-received regular toluene contained 100.8 ppm of water. Regular samples were prepared on the benchtop; care must be taken to minimize exposure of the solvent to the atmosphere by quickly sealing the sample vials.

Wet toluene was prepared by layering toluene (10 ml) over deionized water (1 ml). After shaking and allowing to set for 1 h, wet toluene was carefully withdrawn with a syringe from the top layer without disturbing the lower water phase. Wet toluene prepared with this method contained 343.7 ppm water. Wet samples were prepared on the benchtop; care must be taken to minimize exposure of the solvent to the atmosphere by quickly sealing the sample vials.

UV-vis absorbance measurements were carried out with ca. 10^{-5} M solutions in the solvent specified using a 1 cm quartz cuvette. Emission spectra and fluorescence quantum yields were measured on samples with an optical density of 0.06–0.10 using a 1 cm quartz cuvette.

The samples for *electron microscopy* were prepared in sealed vials at a typical concentration of 1 mM solution (20 to 100 μL) in toluene and left to stand for five minutes. A volume of 5 μL of the formed dispersion was deposited on a continuous carbon 400 mesh C/Cu grid (CF400-Cu-50). The solution was allowed to slowly evaporate under a watch glass. After full evaporation of the solvent, the samples were used for data collection.
The samples for *atomic force microscopy* were prepared from the same solutions used for TEM imaging as described above. 5 μL of solution were deposited on a HOPG (Highly oriented pyrolytic graphite) and allowed to evaporate under a watch glass. After full evaporation of the solvent, the samples were used for AFM imaging.
The samples for *confocal microscopy* were prepared from the same solutions used for TEM imaging as described above. 5 μL of solution were deposited on a glass slide and allowed to evaporate under a watch glass. After full evaporation of the solvent, the samples were used for imaging.

Dynamic light scattering (DLS) measurements were carried out with ca. 600μM dry toluene solutions in the solvent specified using a 1 cm quartz cuvette.

Microscopy Procedures

TEM imaging was performed using a Talos L120C or FEI Tecnai G2 Spirit microscope (Thermo Fisher Scientific, Eindhoven, The Netherlands) equipped with an LaB6 cathode operated at 120 kV acceleration voltage and by using a side-mounted digital camera Gatan Orius 1000 (4k×2.6k pixels, Gatan GmbH, Munich, Germany). For *diffraction*, it was performed using FEI Tecnai G2 Spirit microscope, a selected area aperture of 685 nm image diameter and an instrument camera length of 2.75 m was used.
AFM was performed using a Veeco Nanoscope III to measure the surface morphology in tapping mode. The probe used for the measurement was a HQ:NSC15/Al BS from MikroMasch. The Gwyddion (64 bit) were used to further analyze the AFM images.
Confocal microscopy was performed using a ZEISS LSM 800.

Computational Calculations

Calculations were performed using the Gaussian 16 program package.[1] Ground state geometry optimizations were performed by employing the B3LYP functional,[2–5] the 6-31G(d) basis set[6] and Grimme's D3 dispersion correction[7] with BJ-damping.[8] Frequency calculations at the same level of theory were employed to verify the geometries as local minima possessing no imaginary frequencies. Excited state calculations were carried out within the time-dependent (TD)-DFT approximation, using the same functional, basis set and dispersion correction as above alongside the Solvation Model based on Density (SMD)[9] for solvation using toluene parameters. For every compound, the energetically lowest 30 excited states were calculated. A linewidth of 0.20 eV was assumed for the predicted UV-vis spectra using the Gaussview 6 software.

Solid State Characterization

Crystals suitable for X-ray diffraction were obtained from toluene and MeOH and were mounted on a cryo-loop and used for a low-temperature X-ray structure determination. All measurements were made on a Rigaku Oxford Diffraction XtaLAB Synergy diffractometer with a Pilatus 200 K hybrid pixel area detector using Cu Kα radiation (λ = 1.54184 Å) from a PhotonJet micro-focus X-ray source and an Oxford Cryosystems Cryostream 800 cooler.[10] Data reduction was performed with *CrysAlisPro*.[10] The intensities were corrected for Lorentz and polarization effects, and a numerical absorption correction was applied.[11] The space group was uniquely determined by the systematic absences. Equivalent reflections were merged. The structure was solved by dual space methods using *SHELXT-2018*,[12] which revealed the positions of all non-hydrogen atoms. Neutral atom scattering factors for non-hydrogen atoms were taken from Maslen, Fox and O'Keefe,[13] and the scattering factors for H-atoms were taken from Stewart, Davidson and Simpson.[14]
Samples suitable for X-ray powder diffraction were scratched from the same samples used for confocal microscopy and grinded before measurements. Data were collected on the Rigaku XtaLAB Synergy, Dualflex, HyPix-Arc 150° diffractometer using Cu Kα radiation (λ = 1.54184 Å). 10 images were added for a total collection time of 3330 seconds. The 2D data integration was done in CrysAlisPro.[10]

Micro-electron diffraction

For the 3D electron diffraction (3D ED) measurements, **2H-Car-Ph** nanosheets were deposited on a transmission electron microscopy (TEM) grid. 3D electron diffraction (3D ED) data were collected at room temperature on an ELDICO ED-1 electron diffractometer. The device is equipped with a LaB_6 source operating at an acceleration voltage of 160 kV (λ = 0.02851 Å). For the data collection and initial data assessment, the software Eldix was used. Crystals were mapped and centered in scanning transmission electron microscopy (STEM) imaging mode using a single diode brightfield detector and 3D ED data were collected in diffraction mode (parallel beam, beam size of approximately 700 nm) with a hybrid-pixel detector (Dectris QUADRO). 3D ED data collection was performed under continuous rotation with an angular step of 0.5° per frame and an exposure time of 1 s per frame. Since no significant beam damage was detected during the measurement, data could be collected over a large tilt range (–45° *to +65°).* 3D ED data were

recorded with a diffraction resolution of *ca*. 1 Å. *A total of 13 datasets from 13 individual crystals were collected.* Data processing, evaluation and merging were done using the APEX4 software package.[15] The 3D ED data were indexed, integrated and merged using the APEX4 software package. *Nanosheets of* **2H-Car-Ph** crystallize in a monoclinic lattice (space group No. 15, C2/c) with unit cell constants: a = 33.2(5) Å, *b* = 4.22(6) Å, *c* = 33.0(5) Å, $\alpha = 90°$, $\beta = 106.53(11)°$, $\gamma = 90°$. *Unit cell and space group resulted consistent among all the collected datasets.*
After unit cell determination, the 3D ED data were searched for additional domains and the frames were integrated and corrected for Lorentz effects, scan speed, background and absorption using SAINT[16] and SADABS.[17]
Given the low symmetry space group, data from the four individual crystals diffracting at highest resolution were merged. The resulting completeness (82.1%) was sufficient to allow successful structure determination and stable refinement.
Structure solution from the 3D ED data was carried out using ShelXt,[18,19] and structure refinement was carried out using ShelXL integrated in the ShelXle software.[20] In the refinement calculations, atomic scattering factors and wavelength for electrons were used. Least squares refinement was carried out within the kinematical approximation by minimizing the function $\Sigma w(F_{obs}^2 - F_{calc}^2)^2$ with the ShelXL weighting scheme and using neutral electron scattering factors provided within the ShelXle software. Refinement of non-H atoms was carried out using isotropic atomic displacement parameters, while H atoms were added subsequently with a riding model.
Electron diffraction delivers identical structures to single-crystal X-ray diffraction but with higher statistical values due to dynamic effects. Solutions are refined kinematically and therefore A and B level alerts will always be present. Such values are to be expected from electron diffraction data unlike the data given from single-crystal X-ray diffraction.

Cryo-electron diffraction

Data were collected on a Tecnai G2 sphera equipped with a cheetah D detector from Amsterdam Scientific. The crystal was cooled at liquid nitrogen temperature during the measurement, to decrease radiation damage. Due to the radiation damage, only a limited range was collected on each crystal. Data were integrated in the DIALS software[21] and 11 datasets from individual crystals were merged using dials.cosym. The data could be solved in SHELXD.[22] The structure was refined in SHELXL using the kinematical approximation.

Void and Pore Analysis

The voids can be visualised using the free CCDC crystal structure viewer program Mercury (https://www.ccdc.cam.ac.uk/solutions/software/free-mercury/). Voids were calculated using the standard settings in Mercury (probe radius 1.2 Å, grid spacing 0.3 Å). Pores were analyzed with a He probe with standard settings found in Mercury (σ = 2.58 Å, ε = 10.22 K).

Vapor Annealing Experiments

The samples used for the vapor annealing experiments were obtained using the same procedure as those used for confocal microscopy. The samples were placed in sealed vials under saturated dry/wet toluene vapor. After 12 hours, the glass slides were taken out, and the annealed samples were directly used for further analysis.

Experimental Procedures

3,6-diphenyl-9H-carbazole (**1**)

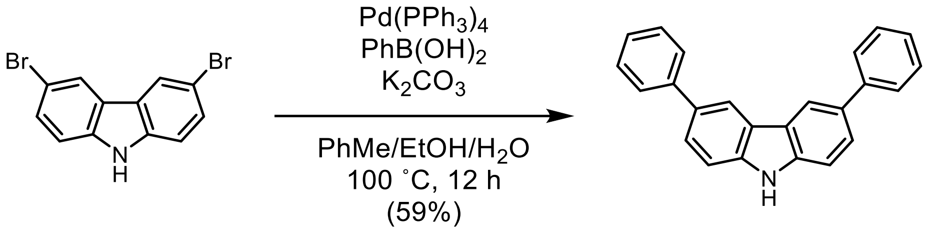

1

A 100 mL round-bottomed flask was charged with 3,6-dibromocarbazole (1.00 g, 3.08 mmol, 1.0 eq.), phenylboronic acid (0.93 g, 7.39 mmol, 2.4 eq.) and potassium carbonate (1.70 g, 12.3 mmol, 4.0 eq.) and toluene (40 mL), ethanol (15 mL) and water (5 mL) were added. N_2 was bubbled through the mixture for 15 minutes and $Pd(PPh_3)_4$ (107 mg, 92 µmol, 3 mol%) was added. The reaction mixture was heated to 100 °C for 12 h before removing solvent *in vacuo*. The crude product was then purified with column chromatography (petroleum ether/CH_2Cl_2, 2:1) to yield **1** (0.58 g, 59%) as a white solid.

Analytical data were in agreement with those reported in literature.[23]

1,8-dibromo-3,6-diphenyl-9H-carbazole (**2**)

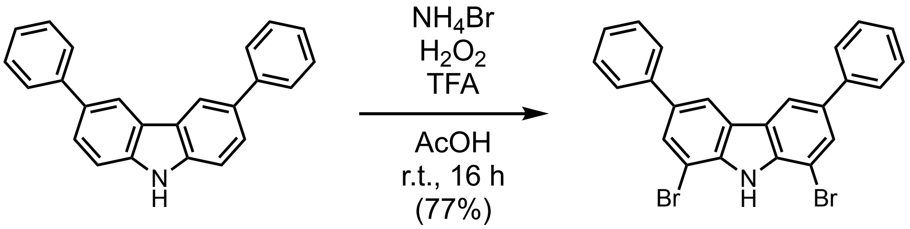

1 **2**

Compound **1** (1.0 g, 3.31 mmol, 1.0 eq.) and NH_4Br (710 mg, 7.26 mmol, 2.2 eq.) were suspended in a mixture of AcOH (15mL) and TFA (3.0 mL). H_2O_2 (30% in H_2O, 0.68 mL, 2.2 eq.) was added dropwise and the mixture was stirred overnight. After neutralization with sat. aq Na_2CO_3, the mixture was extracted with CH_2Cl_2 (2 × 50 mL) and the combined organic extracts were washed with sat. aq $NaHCO_3$ (2 × 20 mL). After drying with Na_2SO_4, evaporation of the solvent, the solid was washed with cold methanol to yield **2** (1.15 g, 77%) as an off-white solid.

^{1}H NMR (400 MHz, $CDCl_3$): δ = 8.32 (s, 1H), 8.19 (d, J = 1.5 Hz, 2H), 7.86 (d, J = 1.5 Hz, 2H), 7.70–7.61 (m, 4H), 7.54–7.42 (m, 5H), 7.42–7.33 (m, 2H) ppm.
^{13}C NMR (101 MHz, $CDCl_3$): δ = 140.63, 137.68, 135.46, 129.07, 128.48, 127.45, 127.34, 125.49, 118.51, 104.90 ppm.
HR-ESI-MS: m/z = 473.95013 [M-H]$^-$ ($C_{24}H_{14}NBr_2^-$ requires 473.94985).

Analytical data were in agreement with those reported in literature.[24]

3,6-diphenyl-1,8-bis(4,4,5,5-tetramethyl-1,3,2-dioxaborolan-2-yl)-9H-carbazole (**3**)

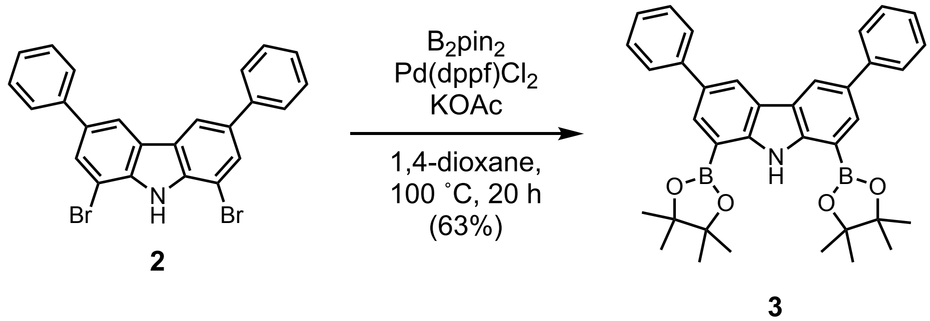

A stirring solution of **2** (314 mg, 0.66 mmol, 1.0 eq.), B_2pin_2 (401 mg, 1.58 mmol, 2.4 eq.) and KOAc (517 mg, 5.26 mmol, 8.0 eq.) in 1,4-dioxane (30 mL) was degassed in a heat-gun dried Schlenk flask by bubbling N_2 through over 15 minutes. $Pd(dppf)Cl_2$ (96 mg, 0.13 mmol, 20 mol%) was added and the reaction mixture was heated to 100 °C for 20 h. The crude product was passed over a silica plug eluted with EtOAc and the solvent was removed *in vacuo*. Purification by column chromatography on silica gel (petroleum ether/CH_2Cl_2, 1:1) yielded **3** (235 mg, 63%) as a white solid.

^{1}H NMR (400 MHz, $CDCl_3$): δ = 10.23 (s, 1H), 8.47 (d, J = 1.9 Hz, 2H), 8.15 (d, J = 1.9 Hz, 2H), 7.81–7.74 (m, 4H), 7.47 (t, J = 7.6 Hz, 4H), 7.38–7.24 (t, J = 7.6 Hz, 2H), 1.50 (s, 24H) ppm.
^{13}C NMR (101 MHz, $CDCl_3$): δ = 145.20, 142.23, 132.44, 132.22, 128.78, 127.50, 126.46, 122.90, 122.35, 84.05, 25.34 (the aromatic C next to the B cannot be observed due to the relaxation time of B) ppm.
HR-ESI-MS: *m/z* = 572.31411 $[M+H]^+$ ($C_{36}H_{40}O_4NB_2^+$ requires 572.31380).

2H-Car-Ph

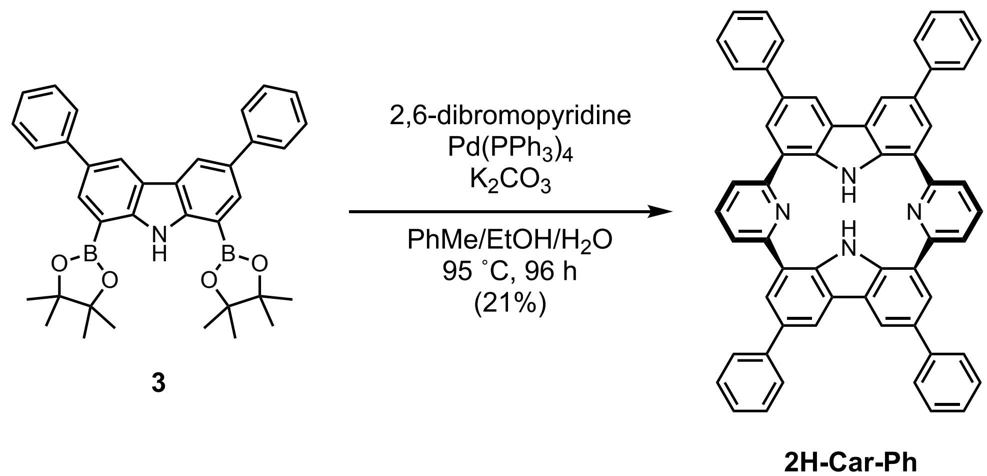

A heat gun-dried 1 L round bottom flask was charged with **3** (112 mg, 0.20 mmol, 1.0 eq.), 2,6-dibromopyridine (23.1 mg, 0.10 mmol, 0.5 eq.) and K_2CO_3 (226 mg, 1.63 mmol, 8.0 eq.) in toluene (50 mL), ethanol (20 mL) and water (7.5 mL). N_2 was bubbled through the biphasic mixture for 15 minutes and $Pd(PPh_3)_4$ (7.1 mg, 6 µmol, 3 mol%) was added. The flask was placed in a pre-heated oil bath and stirred at 85 °C for 96 h. A further 0.5 eq. of 2,6-dibromopyridine was divided into 4 portions and each was added every 12 h. The solvent was removed *in vacuo* and the crude product was dissolved in CH_2Cl_2 (100 mL) and washed with brine (3×50 mL). The organic layer was dried over $MgSO_4$ and the solvent was removed under pressure. The crude mixture was subjected to column chromatography on silica (petroleum ether/CH_2Cl_2, 1:1) before performing recycling gel permeation chromatography ($CHCl_3$). This yielded **2H-Car-Ph** (16.0 mg, 21%) as an off-white solid.

^{1}H NMR (400 MHz, $CDCl_3$): δ = 9.79 (s, 1H), 8.42 (d, J = 1.8 Hz, 4H), 7.99 (t, J = 7.8 Hz, 2H), 7.77–7.72 (m, 12H), 7.66 (d, J = 7.8 Hz, 4H), 7.50 (t, J = 7.7 Hz, 8H), 7.42–7.33 (m, 4H) ppm.
^{13}C NMR (101 MHz, $CDCl_3$): δ = 159.21, 141.75, 138.66, 137.30, 133.92, 128.99, 127.74, 127.48, 126.93, 125.44, 124.60, 122.77, 119.46 ppm.
HR-ESI-MS: *m/z* = 789.30126 $[M+H]^+$ ($C_{58}H_{37}N_4^+$ requires 789.30127).

NMR Characterization

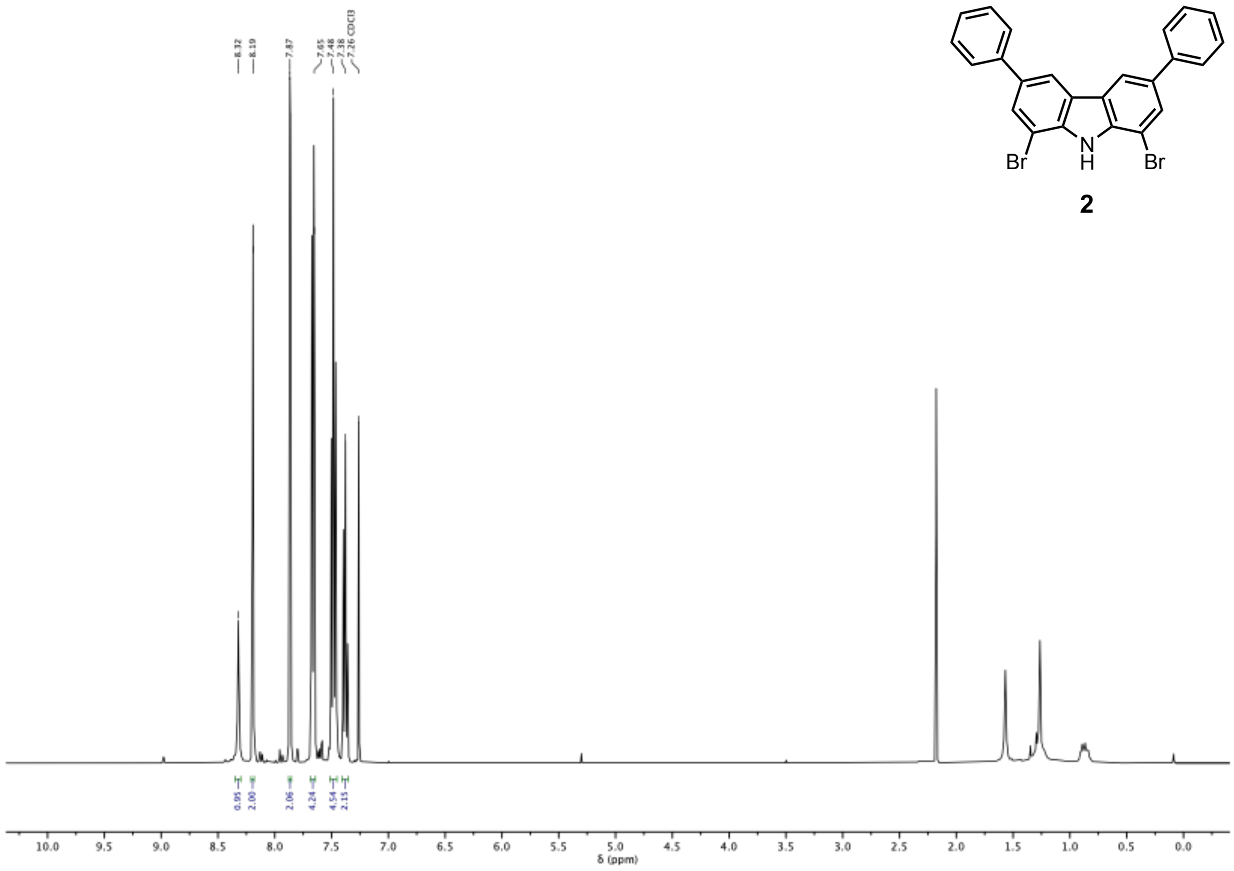

Figure S1. ^{1}H NMR of **2** (400 MHz, 298 K, $CDCl_3$).

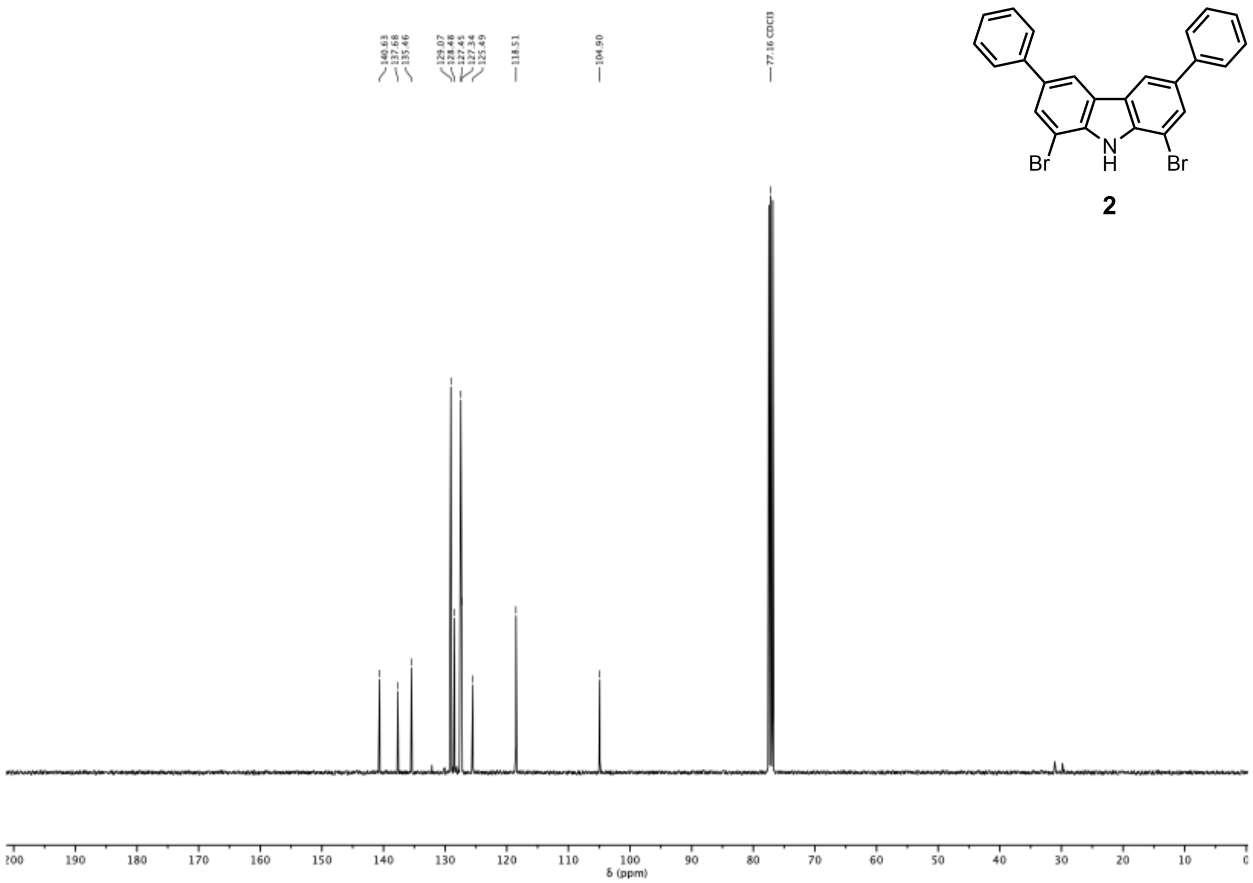

Figure S2. ^{13}C NMR of **2** (101 MHz, 298 K, $CDCl_3$).

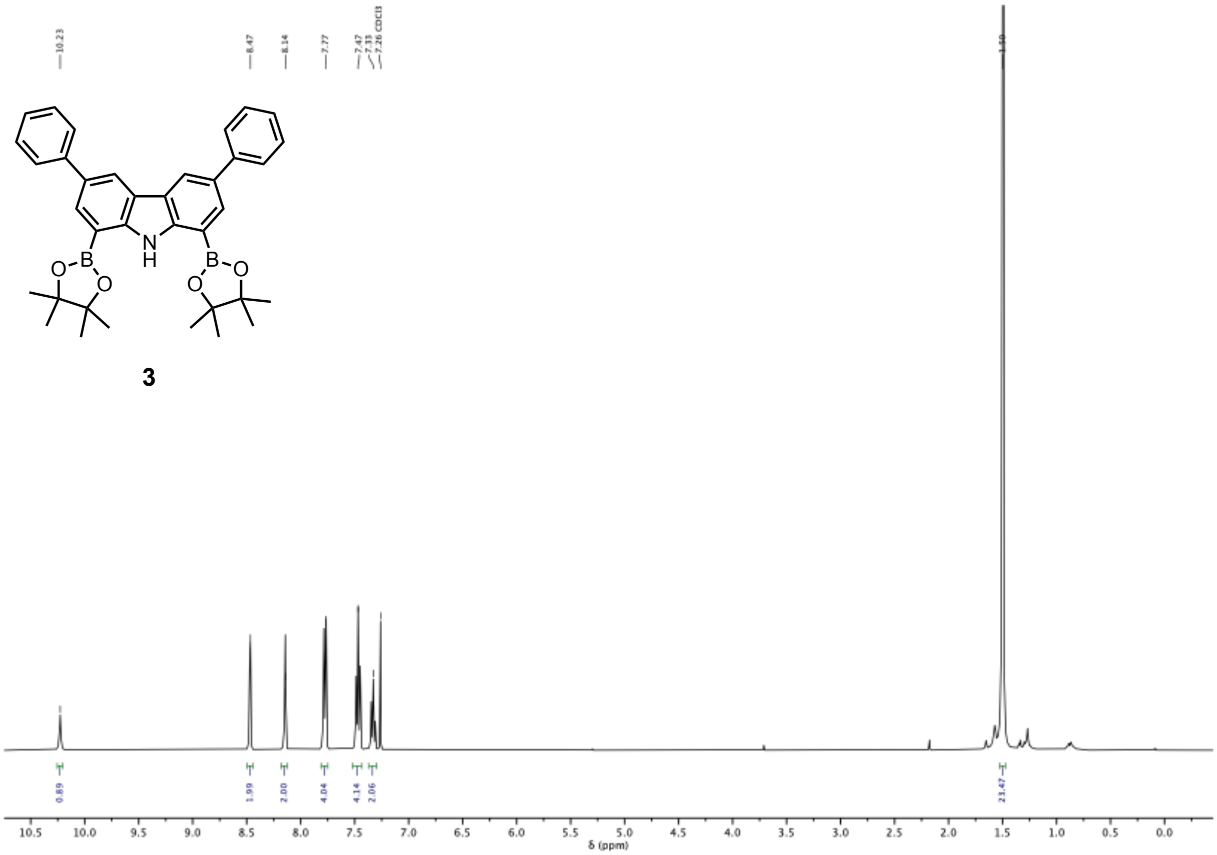

Figure S3. ^{1}H NMR of **3** (400 MHz, 298 K, $CDCl_3$).

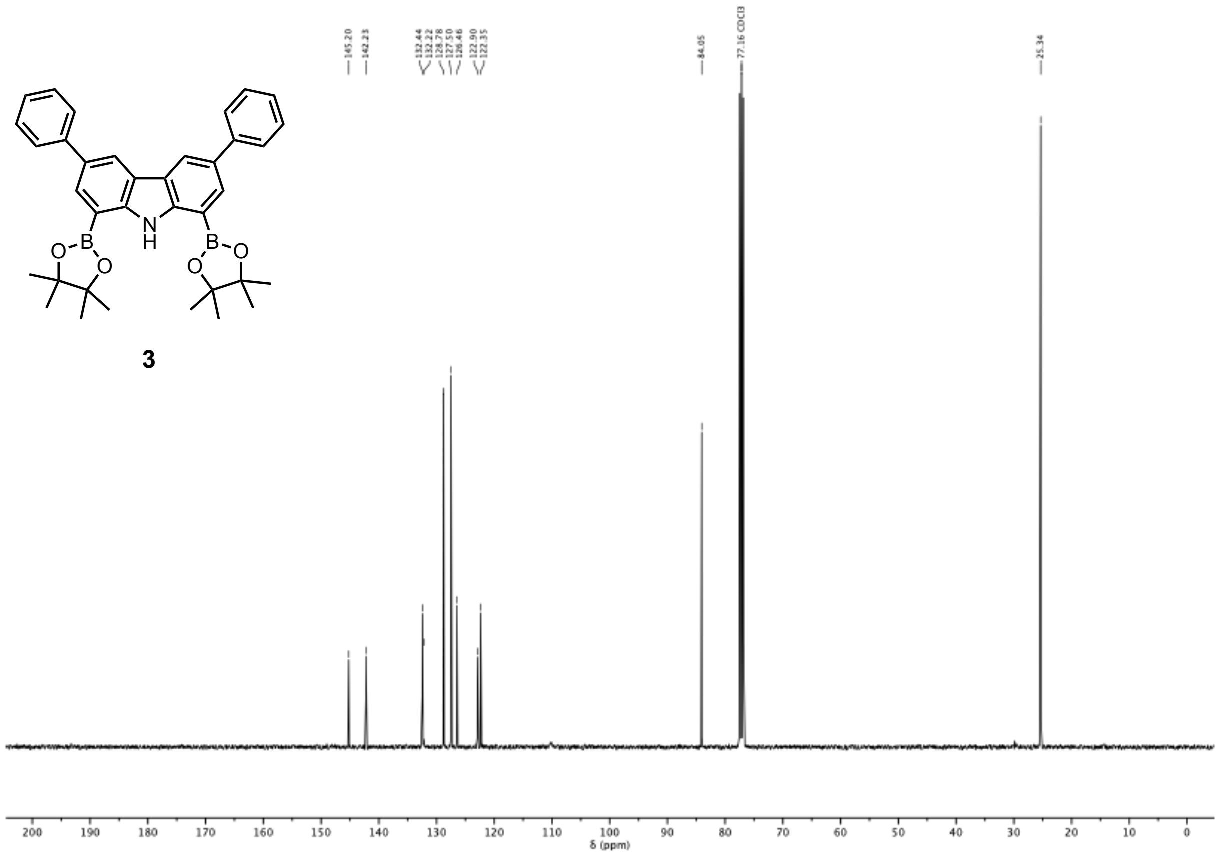

Figure S4. ^{13}C NMR of **3** (101 MHz, 298 K, $CDCl_3$).

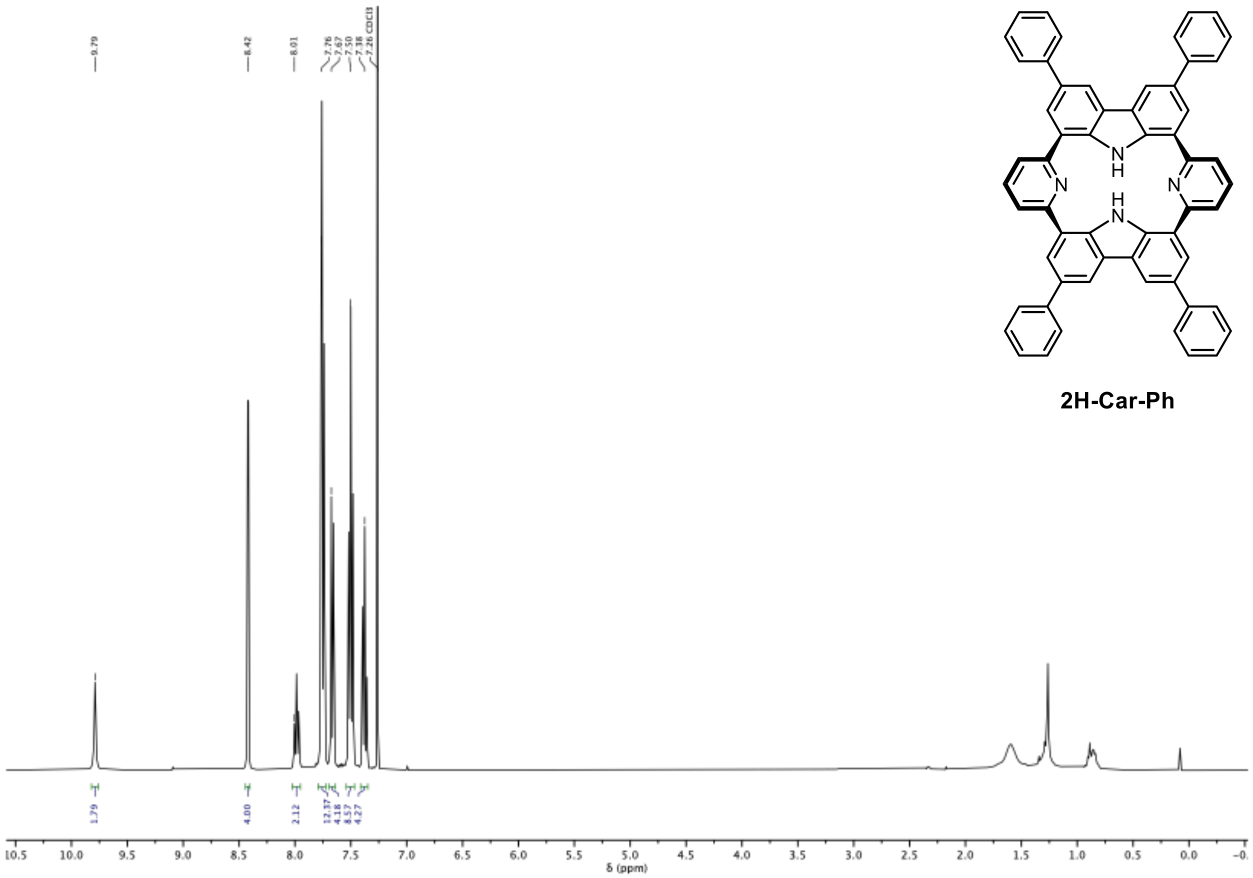

Figure S5. ^{1}H NMR of **2H-Car-Ph** (400 MHz, 298 K, $CDCl_3$).

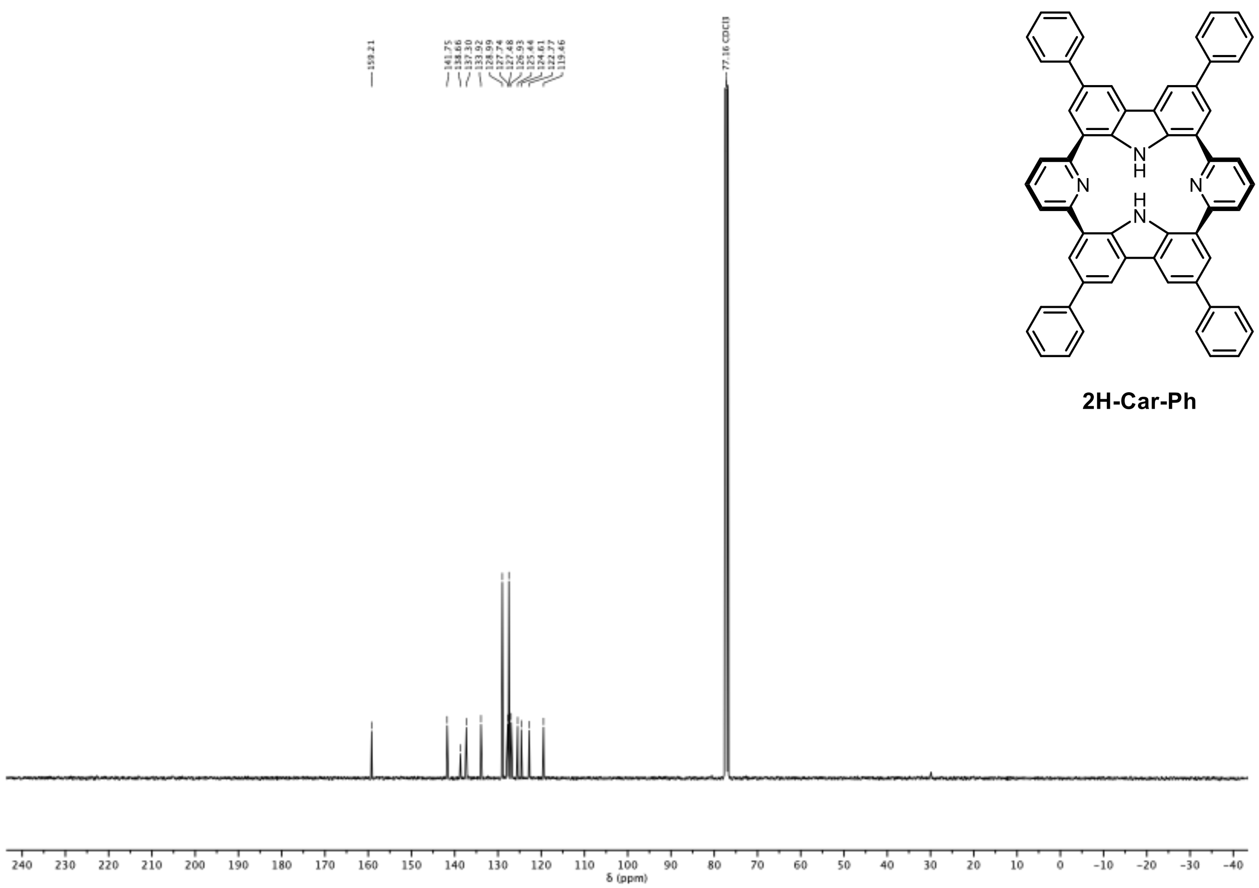

2H-Car-Ph

Figure S6. ^{13}C NMR of **2H-Car-Ph** (101 MHz, 298 K, $CDCl_3$).

Spectroscopic Data

Absorption and emission

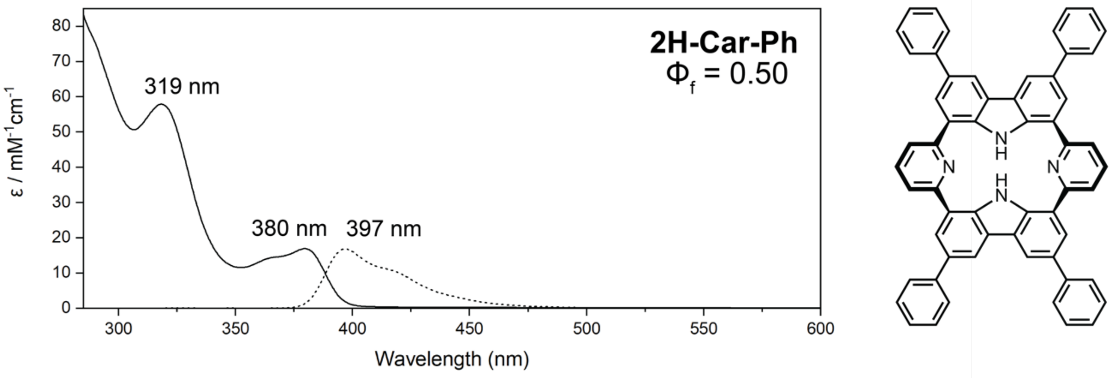

Figure S7. Steady-state UV-vis absorption (continuous lines) and fluorescence emission (dashed lines) spectra of indicated carpyridines. All measurements were made in toluene at 298 K.

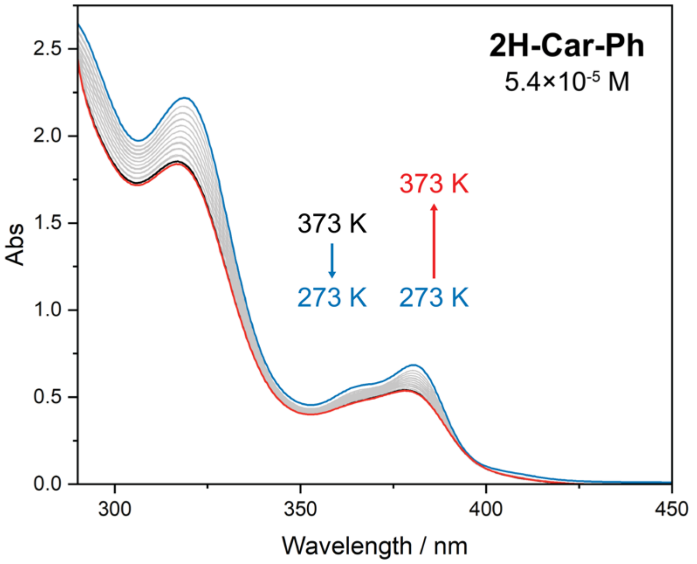

Figure S8. Variable temperature UV-vis (VT-UV) spectra of a 5.4×10^{-5} M solution of **2H-Car-Ph** in dry toluene. The solution was heated to 373 K before slow cooling to 273 K, with spectra measured every 10 K before reheating back to 373 K. Only linear changes due to temperature effects were observed.

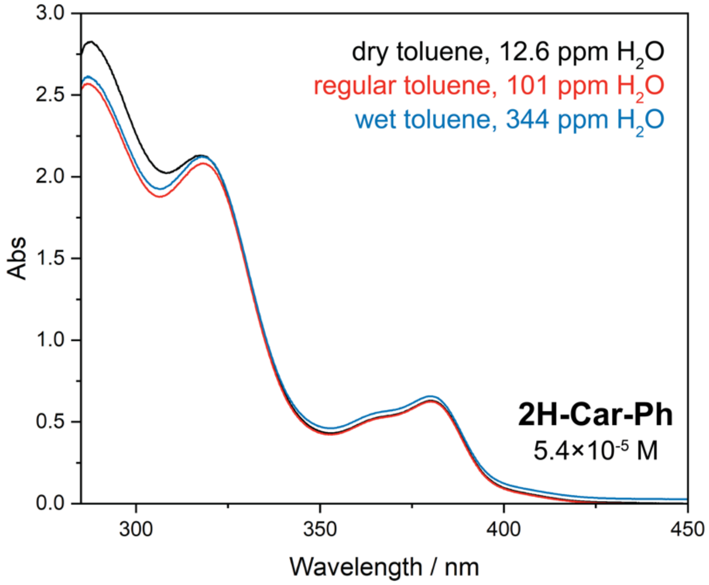

Fig. S9. UV-vis spectra of a 5.4×10^{-5} M solution of **2H-Car-Ph** in toluene with various extents of dryness. No major changes in the absorption spectrum were observed as the water content of the solution changed.

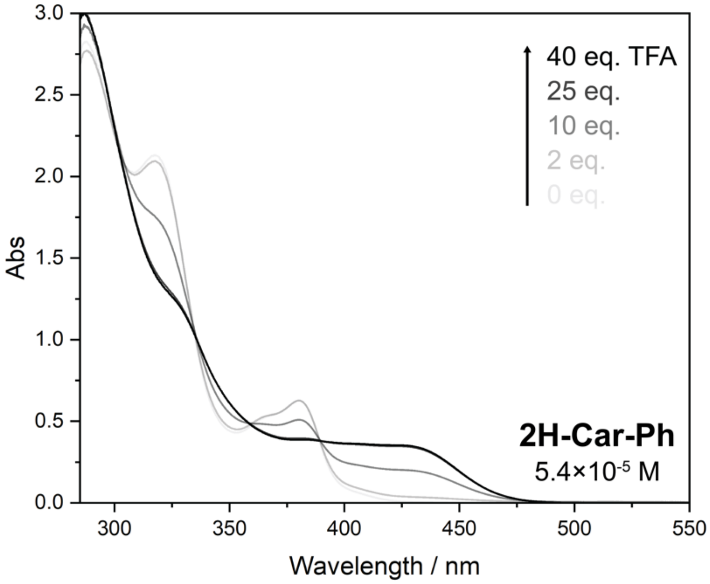

Figure S10. UV-vis spectra of a 5.4×10^{-5} M solution of **2H-Car-Ph** titrated with trifluoroacetic acid (TFA). Upon addition of TFA, protonation of the carpyridine occurs until saturation is reached at 40 eq.

Infra-red

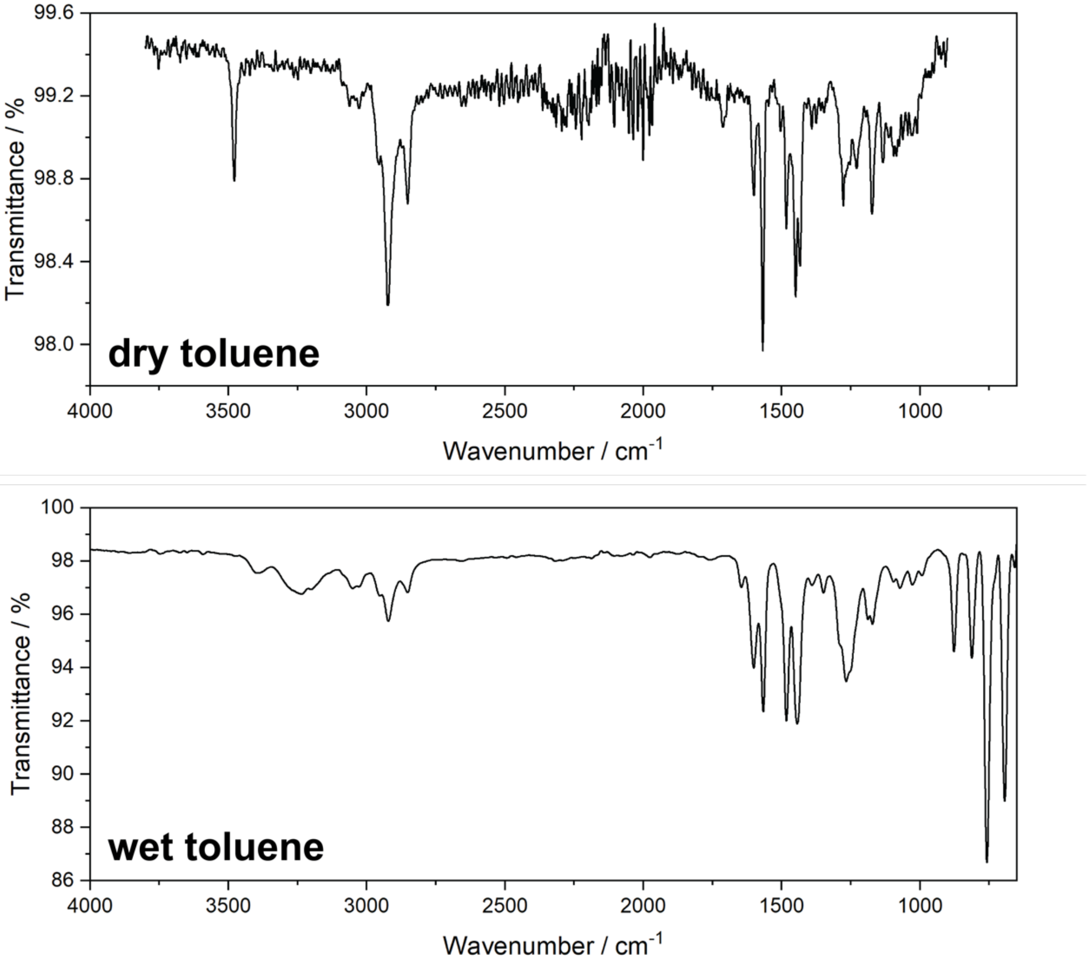

Figure S11. FT-IR spectra of 1 mM **2H-Car-Ph** solutions dropcasted onto the spectrometer from dry toluene, top, and wet toluene, bottom. A single N–H stretch is seen when dropcasted from dry toluene but multiple, broad bands are observed at lower wavenumber when dropcasted from wet toluene.

Microscopy

Additional TEM images

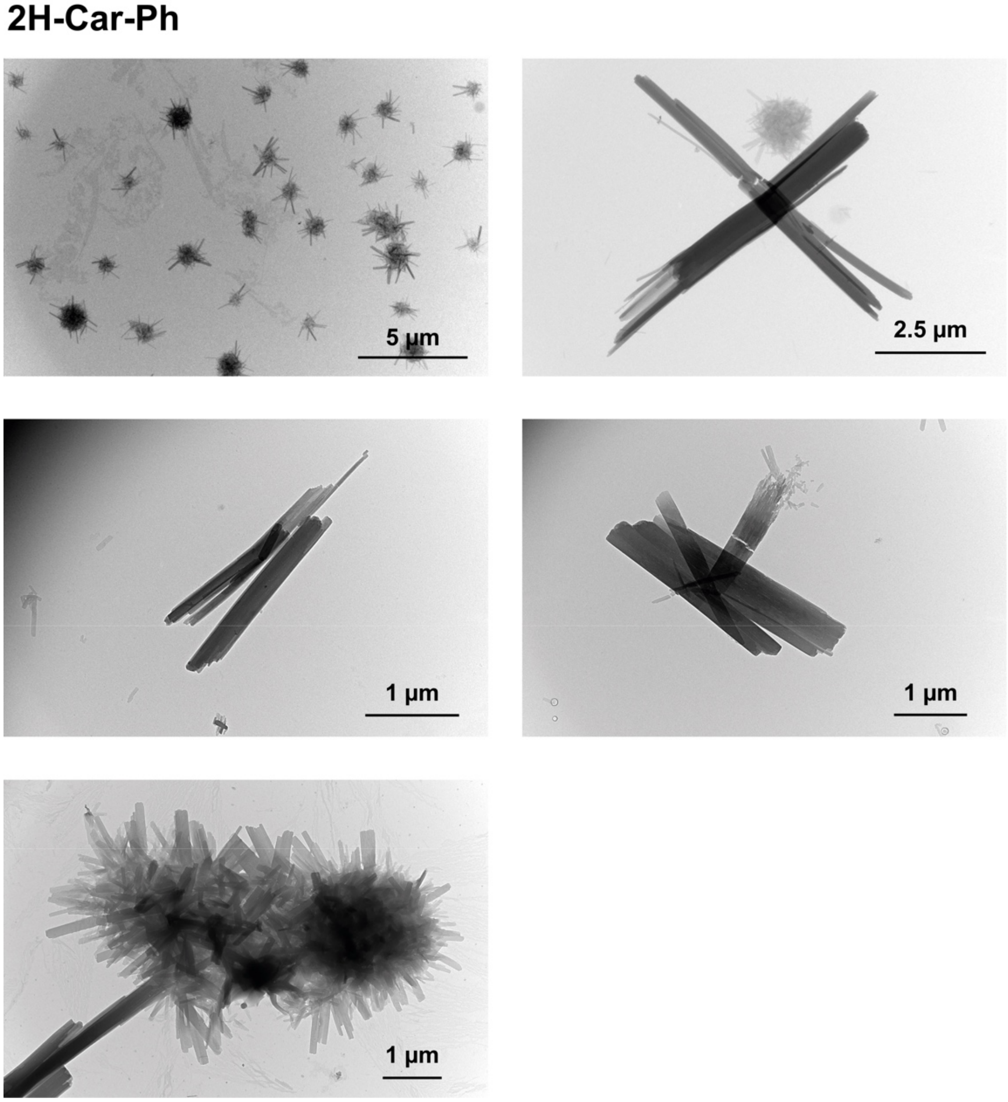

Figure S12. TEM images of **2H-Car-Ph** nanosheets grown from a 1 mM solution in regular analytical grade toluene on a C/Cu grid surface.

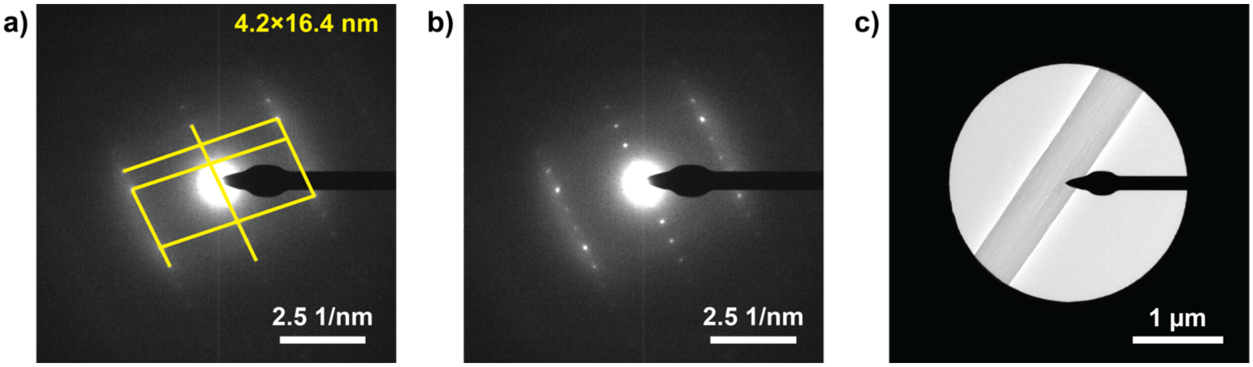

Figure S13. Diffraction patterns obtained from TEM: a) selected area electron diffraction (SAED) pattern of a **2H-Car-Ph** nanosheet taken from a 1 mM regular toluene solution with indicated distances for 2D unit cell parameters. b) the same SAED pattern without annotations and c) the corresponding TEM image of the nanosheet from which the SAED pattern was obtained. Individual diffraction spots were observed, showing the order and crystallinity exemplified by the **2H-Car-Ph** nanosheets. Measuring the distance between two spots in each direction allowed for assignment of a lattice parameter from the single unit cell of the assembly.

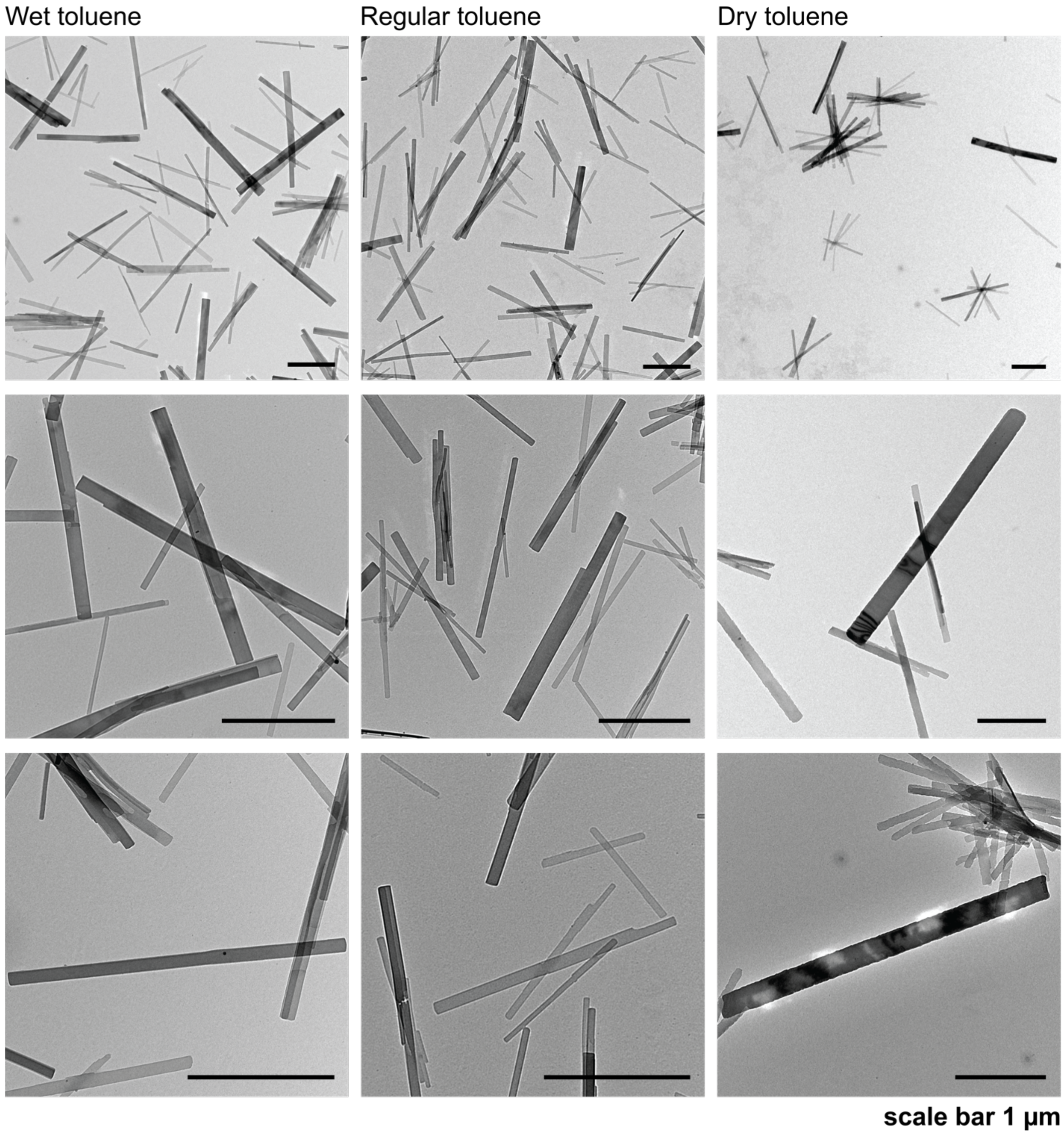

Figure S14. TEM images of **2H-Car-Ph** nanosheets dropcasted from a 1 mM solution in toluene of varying levels of dryness. Wet toluene (344 ppm H_2O), left column, regular analytical grade toluene (101 ppm H_2O), middle column, dry toluene (12.6 ppm H_2O), right column. All solutions formed thin, rectangular nanosheets, however, with increasing water content, increasingly regular nanosheets were observed as can be evidenced by the edge definition. Water content was determined by Karl Fischer titration. Scale bar length corresponds to 1 μm.

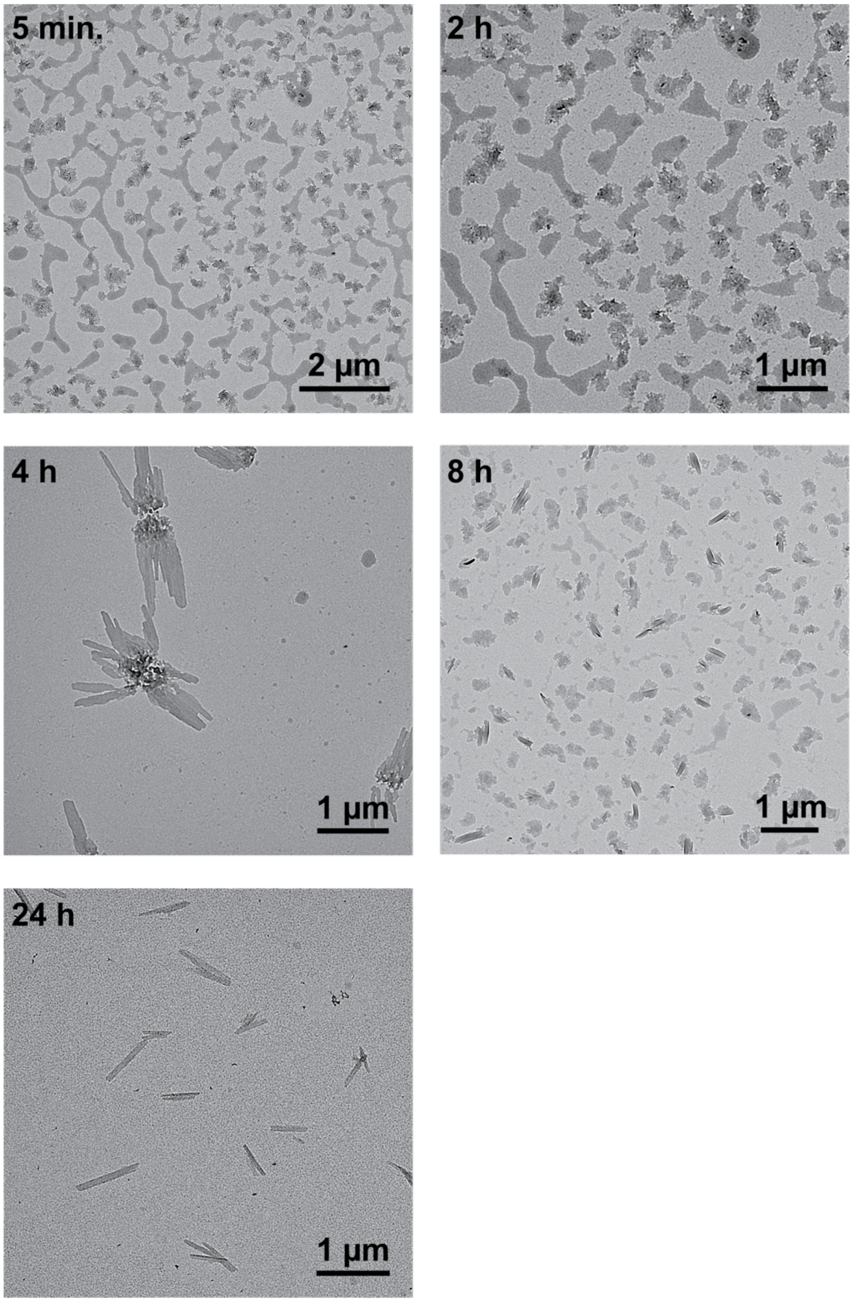

Figure S15. TEM images of **2H-Car-Ph** dropcasted from toluene after heating and allowing the sample to stand at room temperature for various times. Smaller nanosheets were observed after shorter waiting times but larger nanosheets returned after extended durations.

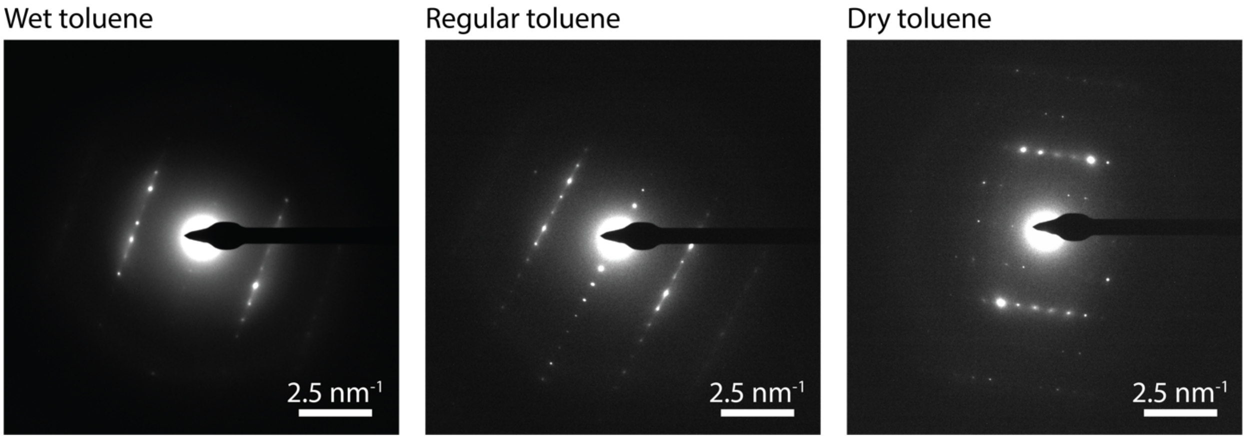

Figure S16. SAED patterns obtained from **2H-Car-Ph** nanosheets when dropcasted from a 1 mM solution in toluene with varying levels of dryness. Wet toluene (344 ppm H_2O), left, regular analytical grade toluene (101 ppm H_2O), middle, dry toluene (12.6 ppm H_2O), right. Wet and regular toluene afforded the same diffraction pattern, however, with dry toluene, nanosheets of a different composition were observed that is reflected in the diffraction pattern. The diffraction pattern obtained from nanosheets under dry conditions was notably more stable under the electron beam that those from regular or wet toluene solutions. For unit cell parameters, see **Table S5**.

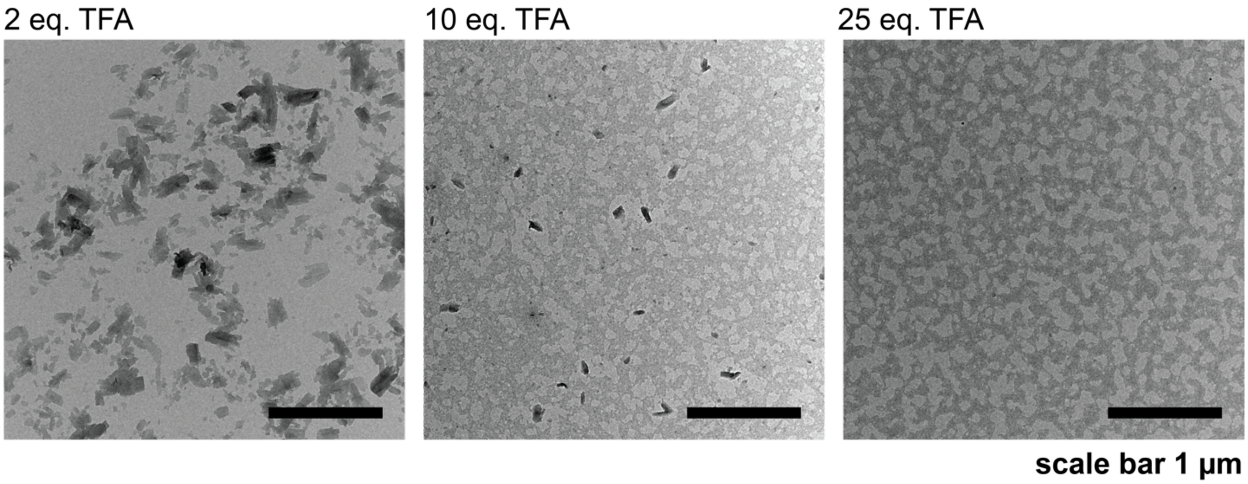

Figure S17. TEM images obtained from a 1 mM solution of **2H-Car-Ph** when dropcasted from toluene with a trifluoroacetic acid (TFA) additive. When 2 equivalents of TFA, left, were added to the 1 mM toluene solution of **2H-Car-Ph**, small flakes of assembled material were seen. Addition of 10 equivalents of TFA, middle, to a 1 mM solution of **2H-Car-Ph** in toluene reduced the number of observable flakes until no flakes were observed when 25 equivalents of TFA, right, were added to a 1 mM solution of **2H-Car-Ph** in toluene. Scale bar length corresponds to 1 µm.

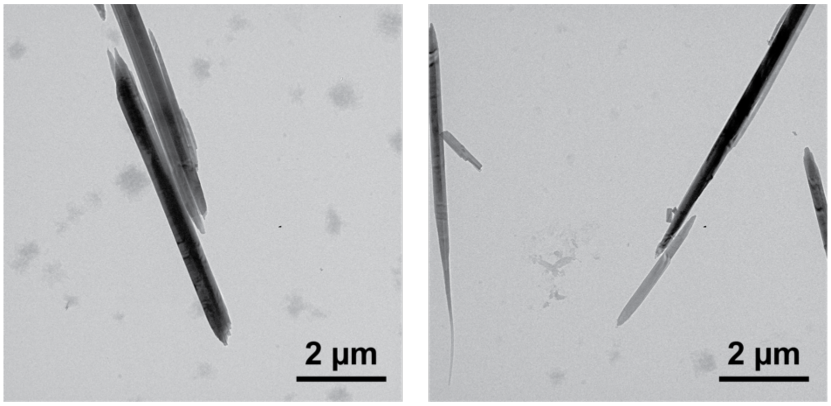

Figure S18. TEM images of 2D nanosheets obtained from a 1 mM dry toluene solution of **2H-Car-Ph** dropcasted onto a glass slide.

AFM images

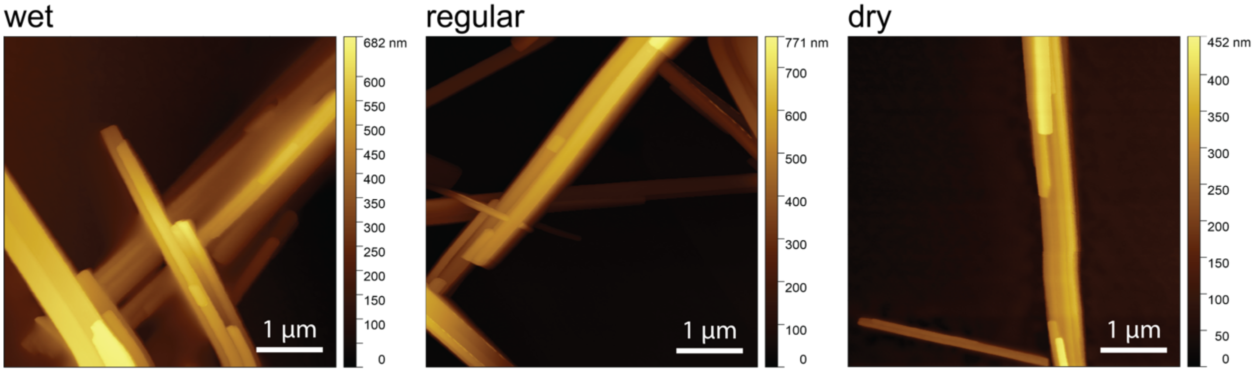

Figure S19. AFM images of **2H-Car-Ph** nanosheets dropcasted from a 1 mM solution of wet (left), regular (middle) and dry (right) toluene on a HOPG surface.

Confocal microscopy

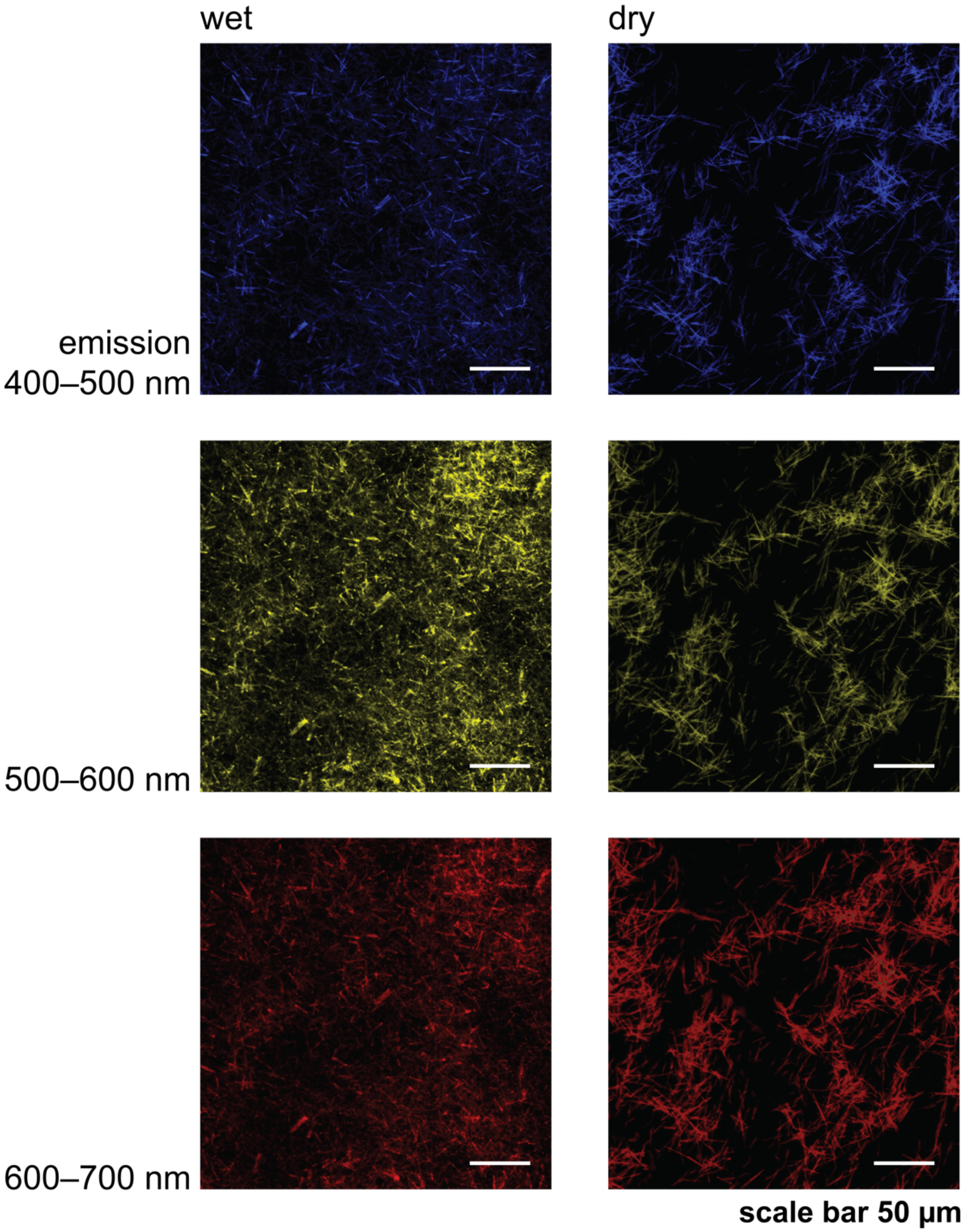

Figure S20. Confocal microscopy images of **2H-Car-Ph** dropcasted from wet and dry 1 mM toluene solutions show emission from nanosheets. Excitation at 405 nm demonstrates fluorescent emission from 400 to 700 nm over three channels for both wet and dry nanosheets. Scale bar for all images is 50 μm.

DFT Calculations

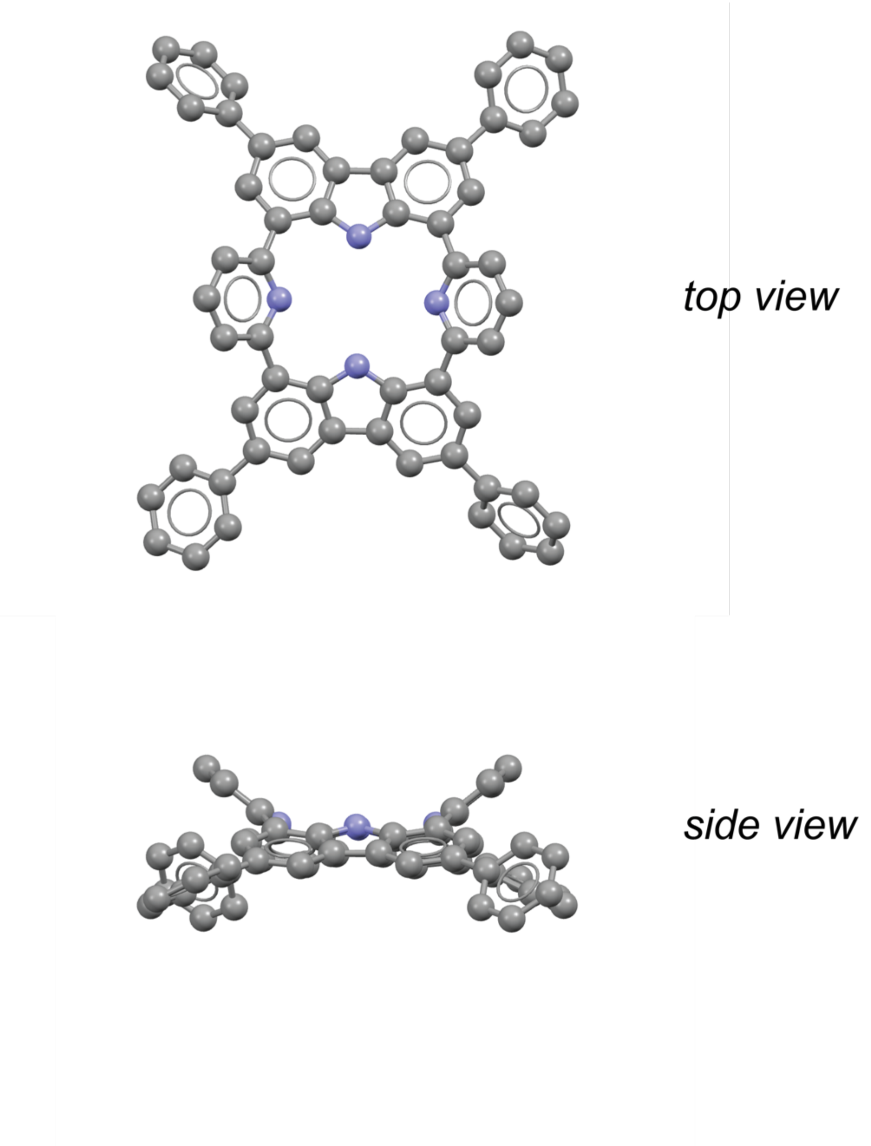

Figure S21. DFT geometry optimization of the **2H-Car-Ph** molecule with B3LYP-(D3BJ)/6-31G(p) basis set. The top and side views are shown, demonstrating the expected saddle topography from the carpyridine core but that the phenyl rings are 37° on average twisted away from the plane of the carbazole. Hydrogen atoms are omitted for clarity.

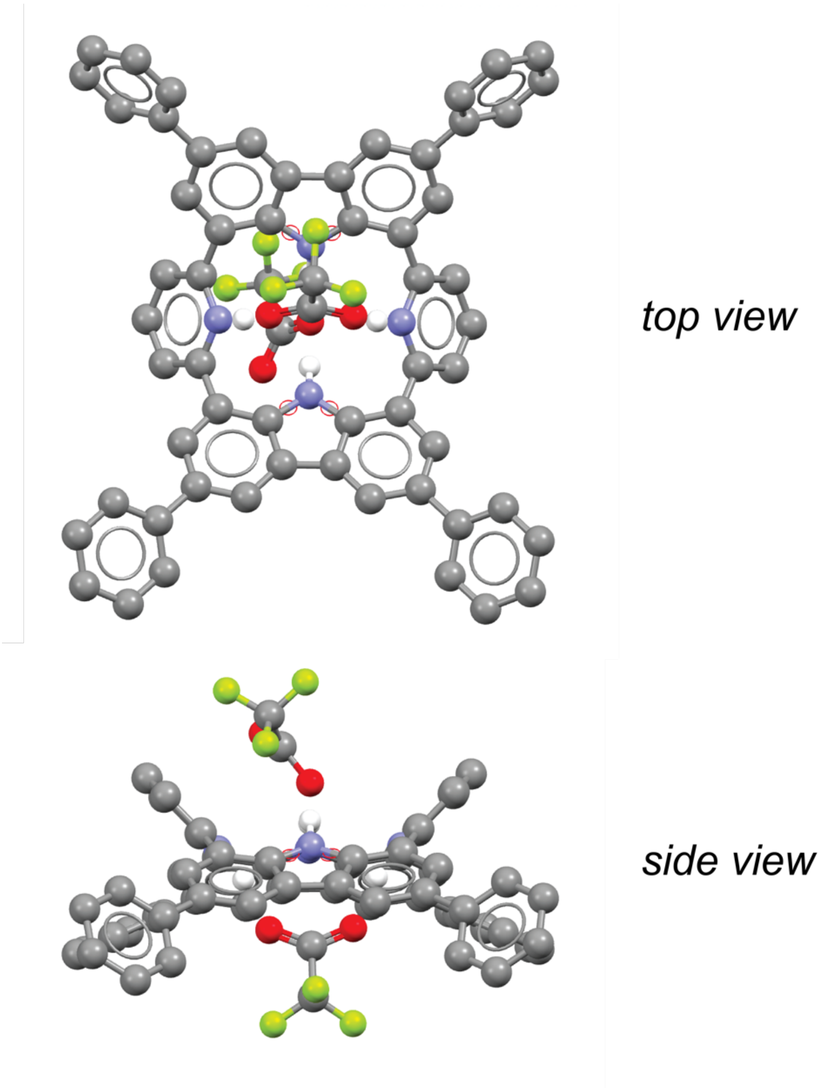

Figure S22. DFT geometry optimization of the **2H-Car-Ph-TFA** complex with B3LYP-(D3BJ)/6-31G(d) basis set. The top and side views are shown, demonstrating the expected saddle topography from the carpyridine core and coordination of the TFA moieties. One TFA molecule lies above the plane of the carpyridine and another below. Hydrogen atoms not interacting with TFA are omitted for clarity.

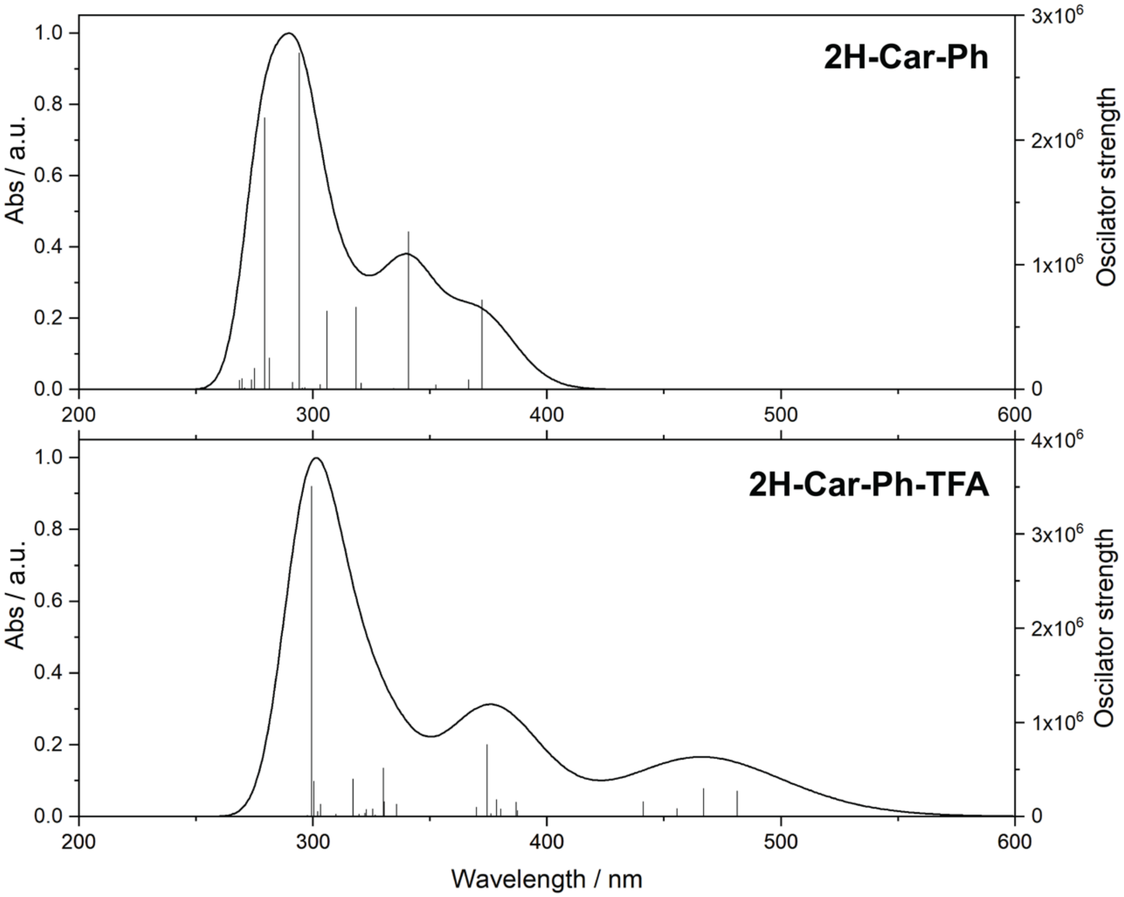

Figure S23. Theoretical UV-vis absorption spectra calculated by TD-DFT (B3LYP(D3BJ)/6-31G(d)) of **2H-Car-Ph** and **2H-Car-Ph-TFA** in toluene with a line width of 0.20 eV. Calculated oscillator strengths are inserted.

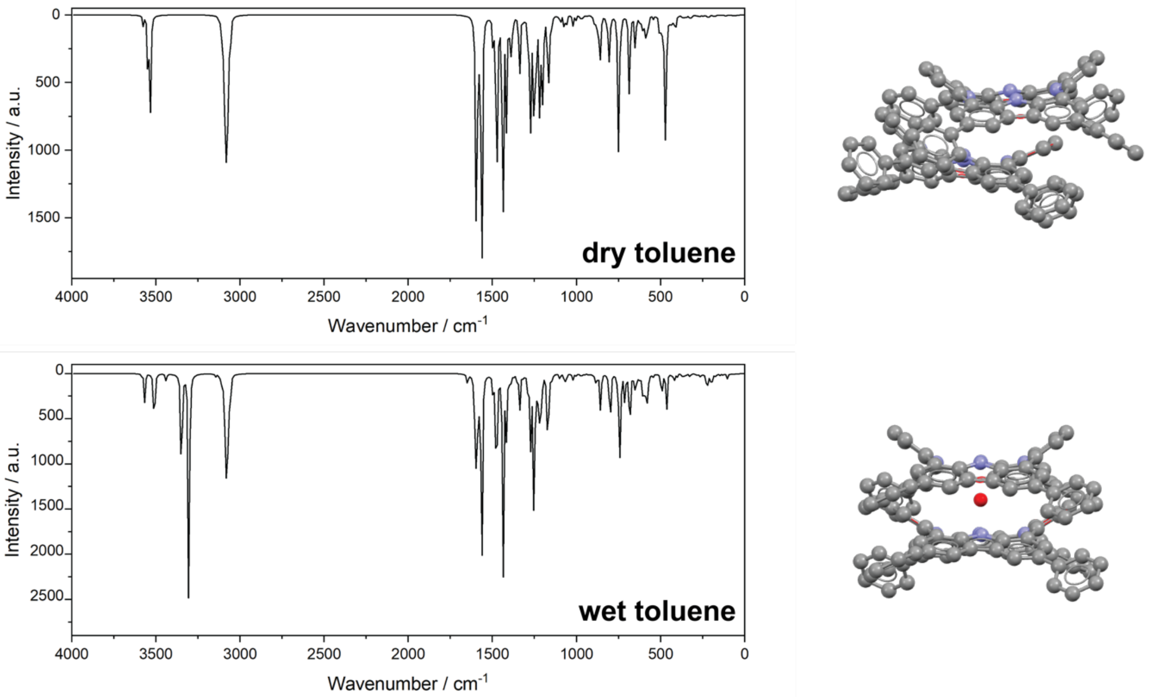

Figure S24. Theoretical IR spectra calculated by DFT (B3LYP(D3BJ)/6-31G(d)) of **2H-Car-Ph** dimer (dry toluene) and **2H-Car-Ph-H_2O** dimer (wet toluene) with a line width of 3 cm^{-1}. Hydrogen atoms are omitted for clarity.

Solid state characterization and analysis

X-ray Diffraction

Definition of Terms

Function minimized: $\mathrm{S}w(F_o^2 - F_c^2)^2$

where $w = [s^2(F_o^2) + (aP)^2 + bP]^{-1}$ and $P = (F_o^2 + 2F_c^2)/3$

$F_o^2 = \mathrm{S(C - RB)/Lp}$

and $s^2(F_o^2) = \mathrm{S^2(C + R^2B)/Lp^2}$

S = Scan rate

C = Total integrated peak count

R = Ratio of scan time to background counting time

B = Total background count

Lp = Lorentz-polarization factor

R-factors: $R_{int} = \mathrm{S}|<F_o^2> - F_o^2| / \mathrm{S}F_o^2$ summed only over reflections for which more than one symmetry equivalent was measured.

$R(F) = \mathrm{S}||F_o| - |F_c|| / \mathrm{S}|F_o|$ summed over all observed reflections.

$wR(F^2) = [\mathrm{S}w(F_o^2 - F_c^2)^2 / \mathrm{S}w(F_o^2)^2]^{1/2}$ summed over all reflections.

Standard deviation of an observation of unit weight (goodness of fit):

$[\mathrm{S}w(F_o^2 - F_c^2)^2 / (N_o - N_v)]^{1/2}$

where N_o = number of observations;

N_v = number of variables

Structure determination for 2H-Car-Ph

Single-crystal X-ray diffraction data were collected at 160(1) K on a Rigaku OD Synergy/Hypix diffractometer using the copper X-ray radiation (l = 1.54184 Å) from a dual wavelength X-ray source and an Oxford Instruments Cryojet XL cooler. The selected suitable single crystal was mounted using polybutene oil on a flexible loop fixed on a goniometer head and immediately transferred to the diffractometer. Pre-experiment, data collection, data reduction and analytical absorption[25] correction were performed with the program suite *CrysAlisPro*.[10] Using *Olex2*[26], the structure was solved with the SHELXT[27] small molecule structure solution program and refined with the *SHELXL2018/3* program package[28] by full-matrix least-squares minimization on F_2. *PLATON* was used to check the result of the X-ray analysis.[29]

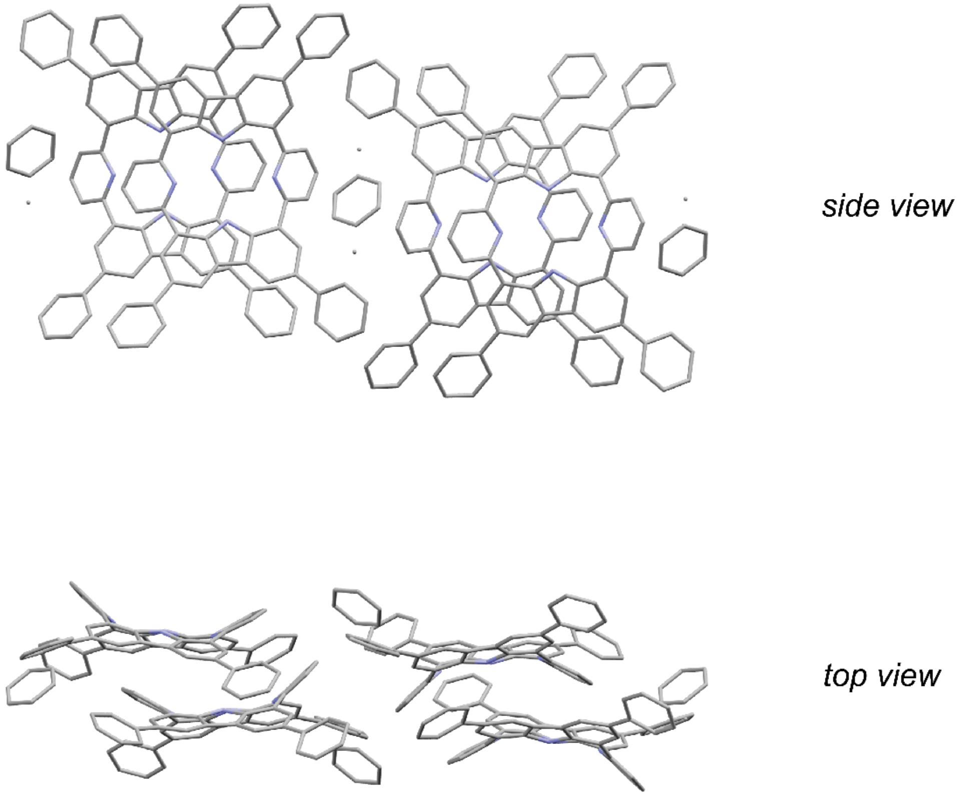

Figure S25. Side and top view from the unit cell of the X-ray structure of **2H-Car-Ph** grown from toluene and MeOH. 4 carypyridine units and 2 toluene solvent molecules are shown.

Table S1. Crystal data and structure refinement for **2H-Car-Ph**.

CCDC No.	2363867
Empirical formula	$C_{123}H_{80}N_8$
Formula weight	1669.95
Temperature/K	160.0(1)
Crystal system	monoclinic
Space group	$P2_1/c$
a/Å	17.5299(3)
b/Å	7.61020(10)
c/Å	31.7286(4)
α/°	90
β/°	94.6650(10)
γ/°	90
Volume/Å^3	4218.77(11)
Z	2
ρ_{calc}g/cm^3	1.315
μ/mm^{-1}	0.592
F(000)	1748.0
Crystal size/mm^3	0.19 × 0.06 × 0.05
Radiation	Cu Kα (λ = 1.54184)
2Θ range for data collection/°	5.058 to 154.76
Index ranges	$-22 \leq h \leq 22$, $-9 \leq k \leq 9$, $-30 \leq l \leq 39$
Reflections collected	51272
Independent reflections	8930 [R_{int} = 0.0227, R_{sigma} = 0.0172]
Data/restraints/parameters	8930/0/604
Goodness-of-fit on F^2	1.043
Final R indexes [I>=2σ (I)]	R_1 = 0.0366, wR_2 = 0.0956
Final R indexes [all data]	R_1 = 0.0407, wR_2 = 0.0992
Largest diff. peak/hole / e Å^{-3}	0.23/-0.25

Structure determination for 2H-Car-Ph-TFA

Suitable crystals were selected and X-ray intensity data were collected on a Rigaku XtaLAB Synergy, Dualflex, HyPix-Arc 150° diffractometer using Cu Kα radiation (λ = 1.54184 Å). Using Olex2,[26] the structures were solved with the SHELXT[27] structure solution program using dual space methods and refined with the SHELXL[28] refinement package using Least Squares minimization. Summaries of crystal data and structure refinement are given in Tables S3 and S4.

The two crystallization conditions (toluene/cyclohexane and $CHCl_3$/cyclohexane) give quasi-identical structure. A model of *C*2/*m* symmetry was refined for the $CHCl_3$/cyclohexane sample. This model could almost fit the data for the sample crystallized in toluene/cyclohexane but a clear violation of the C-centering systematic extinctions was visible (see table S2), so that a model in the space group $P2_1/c$ was preferred. A few crystals were tested on each sample, giving each time the same space-group extinctions.

Table S2. Reciprocal space reconstructions of the hk0 and hk1 plane for the two different crystallization conditions. The projection of the unit-cell is shown in blue. For the CHCl3/cyclohexane, the C-centering extinction (hkl, h+k=2n+1) is fulfilled, whereas violation of this extinction condition appears in the toluene/cyclohexane conditions.

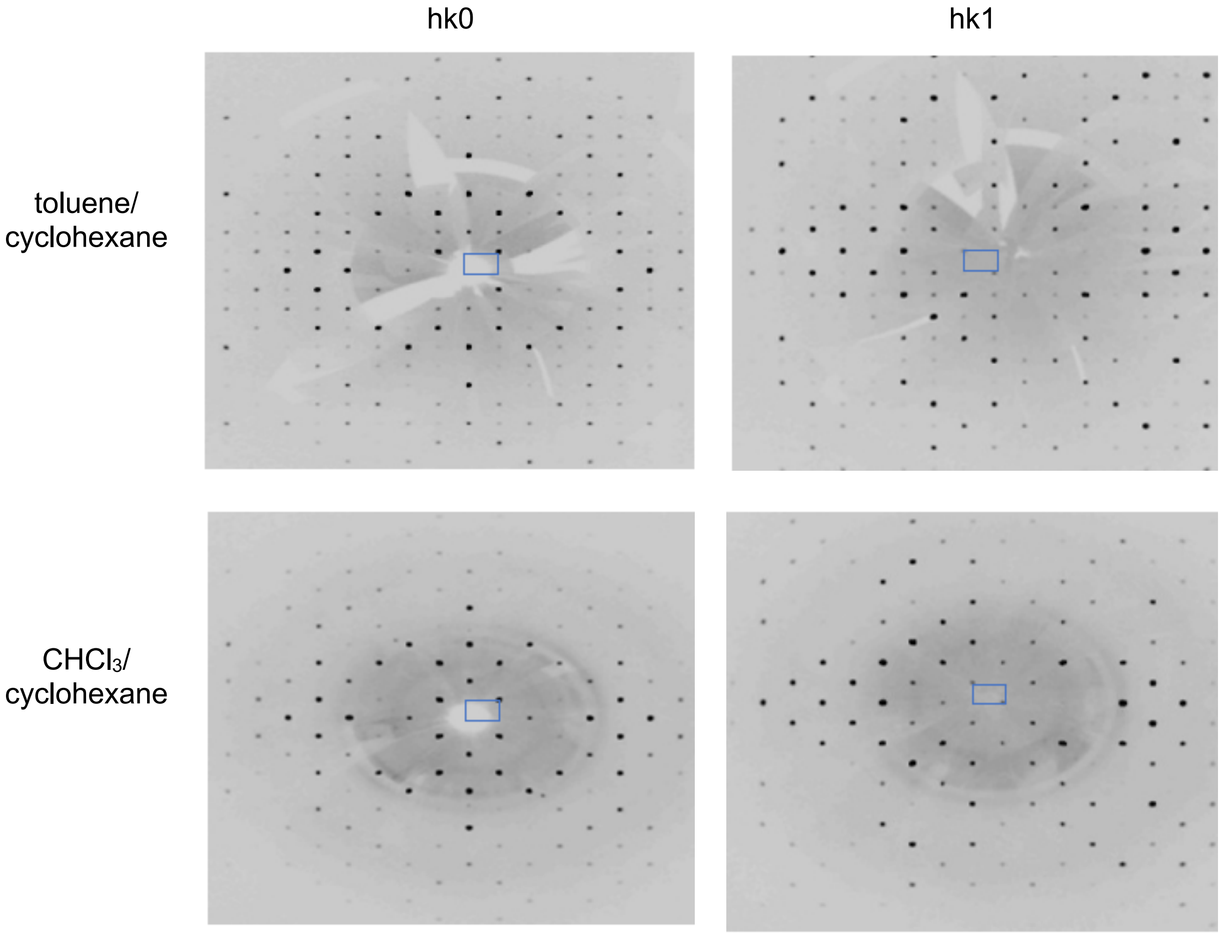

Table S3. Crystal data and structure refinement for **2H-Car-Ph-TFA** crystallized from toluene and cyclohexane.

CCDC No.	2363873	
Empirical formula	C70.33 H49.69 F9 N4 O6	
Formula weight	1217.76	
Temperature	120.00(10) K	
Wavelength	1.54184 Å	
Crystal system	Monoclinic	
Space group	P 1 21/c 1	
Unit cell dimensions	a = 19.2385(2) Å	α = 90°
	b = 18.6164(2) Å	β = 112.1530(10)°
	c = 17.2906(2) Å	γ = 90°
Volume	5735.51(11) Å^3	
Z	4	
Density (calculated)	1.410 Mg/m^3	
Absorption coefficient	0.931 mm^{-1}	
F(000)	2515	
Crystal size	0.349 x 0.026 x 0.015 mm^3	
Theta range for data collection	2.480 to 74.893°.	
Index ranges	$-23 \leqslant h \leqslant 16$, $-23 \leqslant k \leqslant 22$, $-21 \leqslant l \leqslant 21$	
Reflections collected	54309	
Independent reflections	11601 [R(int) = 0.0333]	
Completeness to theta = 67.684°	100.0 %	
Absorption correction	Gaussian	
Max. and min. transmission	1.000 and 0.671	
Refinement method	Full-matrix least-squares on F^2	
Data / restraints / parameters	11601 / 571 / 947	
Goodness-of-fit on F^2	1.066	
Final R indices [I>2sigma(I)]	R1 = 0.0641, wR2 = 0.1678	
R indices (all data)	R1 = 0.0833, wR2 = 0.1806	
Extinction coefficient	n/a	
Largest diff. peak and hole	0.693 and -0.294 e.Å^{-3}	

Comments on the crystals structure determination for the sample crystallized from toluene/cyclohexane:

The proton on the nitrogen were well-visible on the Fourier difference map. They were refined freely.

Two unprotonated TFA molecule hydrogen bonds to the macrocycle. A third TFA molecule is present that has to be protonated for charge balance. It is disordered over two positions and was refined using two components. The following restraints were used:

SADI F8 C75 F7 C75 F9 C75 F10 C77 F11 C77 F12 C77
SADI C75 C74 C77 C76
SADI O8 C74 O7 C76
SADI O6 C74 O5 C76
RIGU C75 O8 C74 O6 F8 F9 F7
RIGU F12 C77 F10 C76 F11 O5 O7
SIMU C77 F12 F10 C76 F11 O5 O7 C75 O8 C74 O6 F8 F9 F7

The assignment of OH group was inferred from a shorter C-O bond length and the possibility for the formation of a hydrogen bond. However, this part is disordered so that the Fourier map and the geometry are not completely unambiguous.

Finally, some remaining density was modelled as a mixed cyclohexane/toluene position with the following restraints applied:

SADI C86 C85 C87 C86 C88 C87 C89 C88 C89 C1E C85 C1E
FLAT C84 C85 C1E C89 C88 C87 C86
RIGU C86 C88 C87 C89 C1E C85 C84
SADI C80 C79 C81 C80 C82 C81 C83 C82 C90 C83 C90 C79
RIGU C80 C83 C90 C82 C79 C81
SIMU C82 C83 C90 C79 C81 C80 C86 C88 C87 C89 C1E C85 C84

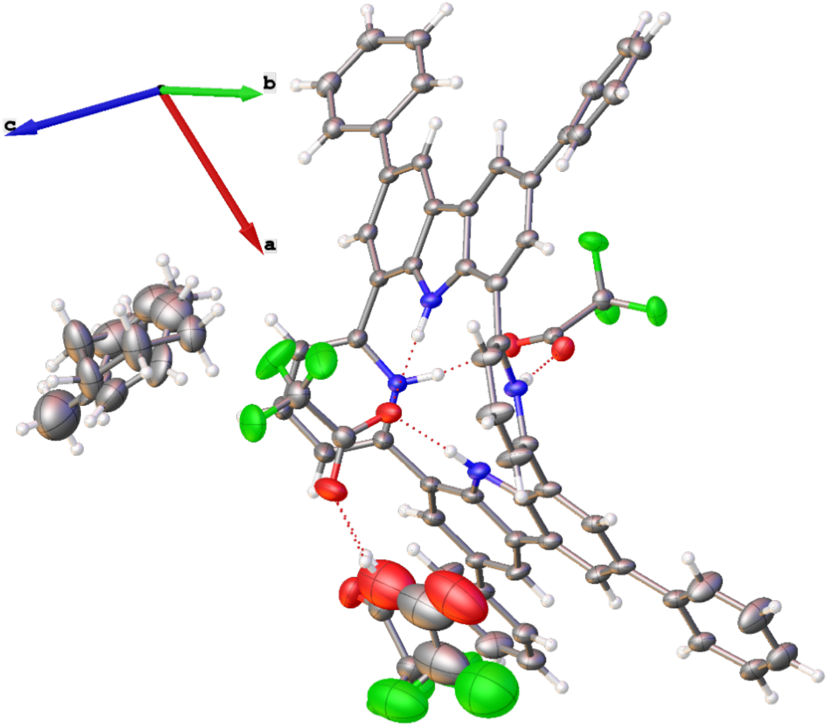

Figure S26. View of the asymmetric unit of the **2H-Car-Ph-TFA** sample crystallized from toluene/cyclohexane. Displacement ellipsoids are depicted at 50 percent probability level.

Table S4. Crystal data and structure refinement for **2H-Car-Ph-TFA** crystallized from $CHCl_3$/cyclohexane.

CCDC No.	2363874	
Empirical formula	C70 H51 F9 N4 O6	
Formula weight	1215.15	
Temperature	119.99(10) K	
Wavelength	1.54184 Å	
Crystal system	Monoclinic	
Space group	C 1 2/m 1	
Unit cell dimensions	a = 17.2907(3) Å	α = 90°
	b = 18.5075(3) Å	β = 112.037(2)°
	c = 19.2410(3) Å	γ = 90°
Volume	5707.42(19) Å^3	
Z	4	
Density (calculated)	1.414 Mg/m^3	
Absorption coefficient	0.934 mm^{-1}	
F(000)	2512	
Crystal size	0.478 x 0.034 x 0.025 mm^3	
Theta range for data collection	2.477 to 75.889°.	
Index ranges	$-21 \leqslant h \leqslant 21, -23 \leqslant k \leqslant 23, -16 \leqslant l \leqslant 23$	
Reflections collected	68894	
Independent reflections	6048 [R(int) = 0.0538]	
Completeness to theta = 67.684°	99.9 %	
Absorption correction	Gaussian	
Max. and min. transmission	1.000 and 0.431	
Refinement method	Full-matrix least-squares on F^2	
Data / restraints / parameters	6048 / 177 / 514	
Goodness-of-fit on F^2	1.030	
Final R indices [I>2sigma(I)]	R1 = 0.0769, wR2 = 0.2186	
R indices (all data)	R1 = 0.0988, wR2 = 0.2407	
Extinction coefficient	n/a	
Largest diff. peak and hole	1.246 and -0.518 e.Å^{-3}	

Comments on the crystals structure determination for the sample crystallized from $CHCl_3$/cyclohexane:

On the macrocycle, there are two C6 rings that are slightly disordered and that were refined using two components. The following restraints/constraints were used:

RIGU C21 C22B C23B C24B C25
RIGU C21 C22 C23 C24 C25
RIGU C22B C22 C23 C23B C24 C24B

RIGU C5 C4B C3B C2B C1
RIGU C5 C4 C3 C2 C1
SIMU C4 C4B C3 C3B C2 C2B

One protonated TFA was refined using the following restraints:

SIMU F5 F5 F6 F6 F7 F7 C54 C54
SADI F6 C54 F7 C54 F5 C54
SADI F7 F6 F6 F5 F5 F7

The H bond to N atoms were refined freely. The position of the H on the protonated TFA was inferred based on the bond length and the potential H bonds.

One cyclohexane solvent molecule was included in the model. This solvent part may be furthered disordered, as indicated by small residual density around. Restraints were used for this molecule:

SADI C37 C30 C33 C30 C34 C33

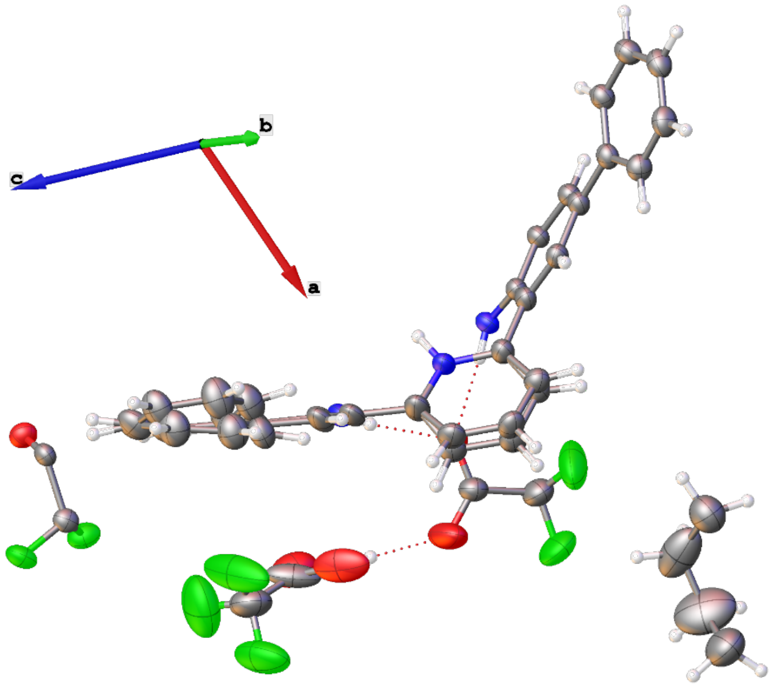

Figure S27. View of the asymmetric unit of the **2H-Car-Ph-TFA** sample crystallized from $CHCl_3$/Cyclohexane. Displacement ellipsoids are depicted at 50 percent probability level.

Micro-electron diffraction

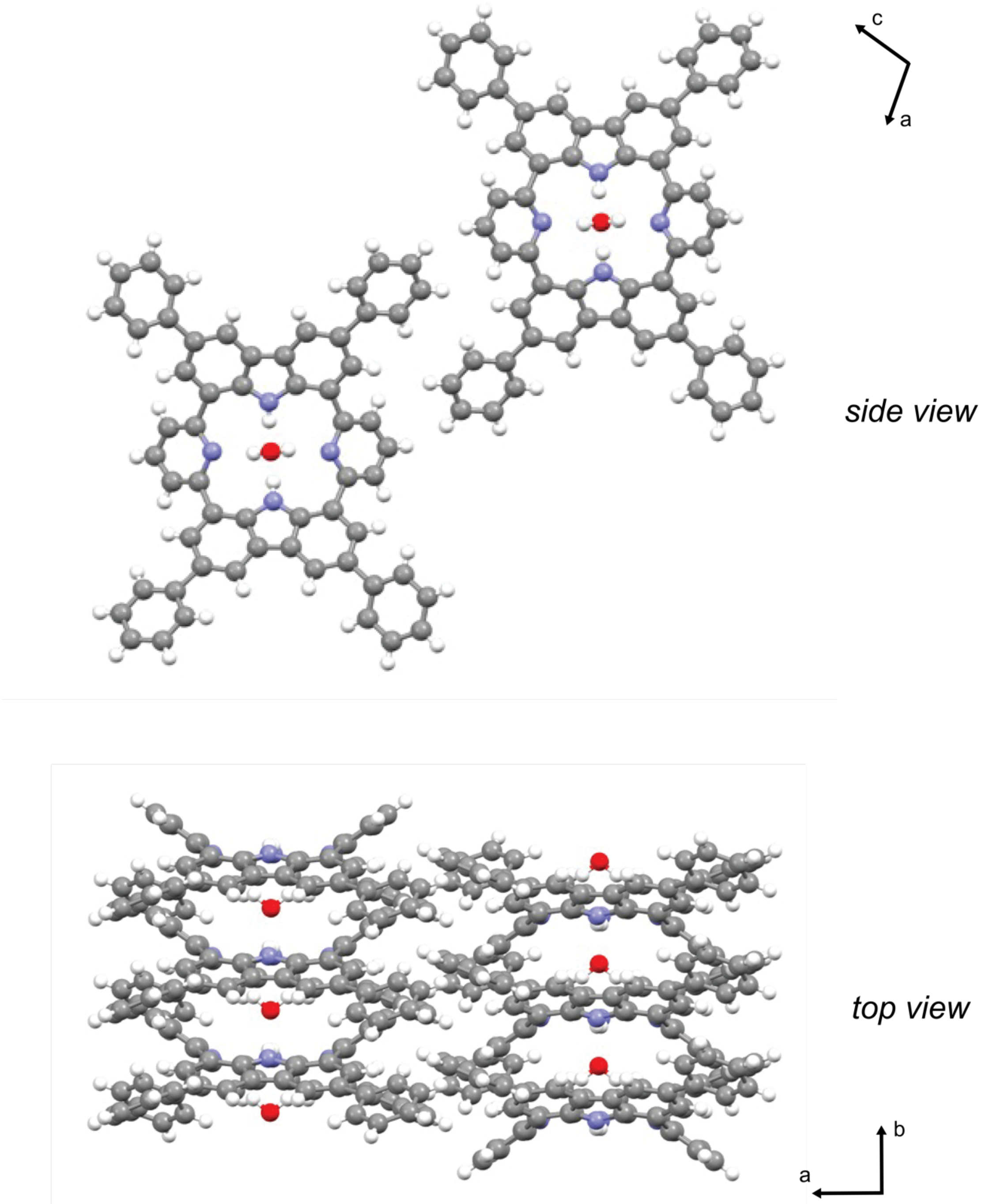

Figure S28. Side view and top view of the micro-electron diffraction (μ-ED, r.t.) structure of **2H-Car-Ph** water-containing nanosheets when dropcasted from a 1 mM solution in regular analytical grade toluene.

Table S5. Crystal data and structural refinement for the room temperature micro-ED measurement of **2H-Car-Ph**.

Identification code	2H-Car-Ph
CCDC No.	2363869
Empirical formula	$C_{58}H_{38}N_4O$
Formula weight	806.92
Temperature/K	273.15
Crystal system	monoclinic
Space group	C2/c
a/Å	33.2(5)
b/Å	4.22(6)
c/Å	33.0(5)
α/°	90
β/°	106.53(11)
γ/°	90
Volume/Å^3	4436(110)
Z	4
ρ_{calc}g/cm^3	1.208
μ/mm^{-1}	0.000
F(000)	706.0
Radiation	electrons (λ = 0.02851)
2Θ range for data collection/°	0.206 to 1.648
Index ranges	$-32 \leq h \leq 32$, $-4 \leq k \leq 4$, $-33 \leq l \leq 33$
Reflections collected	15713
Independent reflections	1831 [R_{int} = 0.3474, R_{sigma} = 0.3155]
Data/restraints/parameters	1831/327/286
Goodness-of-fit on F^2	1.643
Final R indexes [I>=2σ (I)]	R_1 = 0.3163, wR_2 = 0.5408
Final R indexes [all data]	R_1 = 0.3651, wR_2 = 0.5772
Largest diff. peak/hole / e Å^{-3}	0.19/-0.14

Table S6. Fractional Atomic Coordinates (×10[4]) and Equivalent Isotropic Displacement Parameters ($Å^2×10^3$) for **2H-Car-Ph**. U_{eq} is defined as 1/3 of the trace of the orthogonalised U_{IJ} tensor.

Atom	*x*	*y*	*z*	*U(eq)*
C2	6069(4)	7660(40)	8021(7)	81(4)
C3	6485(4)	6850(40)	8222(7)	88(4)
C4	6731(5)	5320(40)	7998(7)	92(4)
C5	6566(4)	4950(40)	7571(7)	91(4)
C6	6164(4)	5950(40)	7362(7)	86(4)
C7	5907(5)	5810(40)	6917(9)	80(4)
C8	5512(6)	7250(40)	6891(10)	79(4)
O1	5000	12890(60)	7500	98(13)
C9	5209(6)	7550(40)	6541(10)	78(4)
C10	5300(6)	6650(40)	6181(9)	87(4)
C11	5993(6)	5000(40)	6567(9)	84(4)
C12	5684(6)	5170(50)	6136(10)	90(4)
C13	5815(6)	4460(50)	5823(9)	102(4)
C14	5554(7)	5020(60)	5416(8)	115(5)
C15	5652(6)	4590(60)	5034(9)	125(5)
C16	5962(7)	2580(50)	5002(11)	122(5)
C17	6225(8)	1810(60)	5390(9)	117(5)
C18	6117(6)	2470(50)	5754(9)	109(5)
C19	7159(5)	4440(50)	8204(9)	106(4)
C20	7288(5)	3210(50)	8619(9)	115(5)
C21	7992(6)	2680(50)	8628(9)	118(5)
C22	7876(5)	3680(60)	8216(9)	117(5)
C23	7473(4)	4510(50)	8000(8)	113(5)
C24	7701(5)	2350(50)	8848(9)	121(5)
C25	5827(6)	9030(50)	8324(9)	87(4)
C26	6018(6)	10750(50)	8664(10)	90(4)
C27	5824(6)	11820(50)	8933(10)	93(5)
C28	5400(6)	10740(50)	8851(10)	90(5)
C29	5207(6)	8880(50)	8504(10)	88(5)
N1	5426(5)	7930(40)	8245(8)	81(4)
C1	5904(5)	7240(40)	7591(7)	80(4)
N2	5536(5)	7920(30)	7337(8)	80(4)

Table S7. Anisotropic Displacement Parameters ($Å^2 \times 10^3$) for **2H-Car-Ph**. The Anisotropic displacement factor exponent takes the form: $-2\pi^2[h^2a^{*2}U_{11}+2hka^*b^*U_{12}+…]$.

Atom	U_{11}	U_{22}	U_{33}	U_{23}	U_{13}	U_{12}
C2	43(5)	111(7)	104(8)	5(7)	44(6)	-4(5)
C3	44(6)	122(8)	110(9)	6(8)	39(6)	-4(6)
C4	43(5)	125(7)	117(9)	9(8)	39(6)	-2(5)
C5	45(6)	122(8)	115(9)	3(8)	39(6)	-3(6)
C6	41(5)	121(7)	108(8)	1(7)	44(5)	-5(5)
C7	34(5)	116(7)	107(8)	-1(7)	49(5)	0(5)
C8	34(5)	115(7)	104(8)	-2(7)	44(6)	2(5)
O1	83(18)	121(18)	90(40)	0	30(30)	0
C9	33(6)	106(8)	106(10)	-2(7)	38(6)	-2(6)
C10	44(6)	121(8)	109(9)	-4(8)	39(6)	-6(6)
C11	44(6)	114(8)	109(9)	-11(8)	47(6)	4(6)
C12	46(6)	122(7)	113(9)	-13(7)	40(6)	3(6)
C13	63(8)	144(8)	111(10)	-14(8)	46(7)	-1(6)
C14	76(9)	161(10)	114(10)	-20(10)	37(8)	3(7)
C15	92(9)	173(10)	112(11)	-18(10)	30(9)	3(8)
C16	86(10)	177(11)	110(12)	-20(10)	39(9)	0(8)
C17	83(9)	169(10)	109(13)	-23(10)	44(9)	11(8)
C18	73(8)	157(10)	112(12)	-18(9)	47(8)	7(7)
C19	45(5)	147(8)	131(11)	18(8)	34(6)	7(6)
C20	52(6)	159(10)	137(12)	27(9)	33(8)	0(7)
C21	46(7)	164(10)	144(14)	22(10)	25(8)	4(7)
C22	47(6)	167(10)	138(13)	11(10)	29(9)	5(7)
C23	45(6)	165(10)	133(12)	10(9)	33(8)	7(7)
C24	55(7)	164(10)	142(13)	28(10)	28(8)	3(7)
C25	51(6)	119(7)	100(9)	-4(7)	38(7)	1(5)
C26	55(7)	123(9)	102(11)	-3(7)	36(8)	-3(6)
C27	60(7)	130(9)	99(11)	-7(8)	37(8)	5(6)
C28	59(7)	125(9)	96(12)	-5(8)	38(8)	11(6)
C29	57(7)	124(9)	94(12)	-1(8)	38(8)	5(6)
N1	46(6)	116(8)	93(11)	4(7)	41(7)	2(5)
C1	42(5)	112(7)	101(8)	5(7)	46(6)	-2(5)
N2	42(5)	117(7)	99(9)	3(7)	48(6)	3(5)

Table S8. Bond Lengths for **2H-Car-Ph**.

Atom	*Atom*	*Length/Å*	*Atom*	*Atom*	*Length/Å*
C2	C3	1.39(2)	C13	C18	1.38(2)
C2	C25	1.56(3)	C14	C15	1.40(3)
C2	C1	1.38(3)	C15	C16	1.36(2)
C3	C4	1.41(2)	C16	C17	1.37(3)
C4	C5	1.37(3)	C17	C18	1.37(3)
C4	C19	1.44(3)	C19	C20	1.41(3)
C5	C6	1.38(2)	C19	C23	1.39(2)
C6	C7	1.48(3)	C20	C24	1.41(2)
C6	C1	1.41(2)	C21	C22	1.37(3)
C7	C8	1.42(3)	C21	C24	1.37(2)
C7	C11	1.31(4)	C22	C23	1.37(2)
C8	C9	1.31(4)	C25	C26	1.34(4)
C8	N2	1.48(4)	C25	N1	1.36(3)
C9	C10	1.36(4)	C26	C27	1.32(3)
C9	C29[1]	1.46(3)	C27	C28	1.43(3)
C10	C12	1.46(3)	C28	C29	1.38(4)
C11	C12	1.50(4)	C29	N1	1.33(3)
C12	C13	1.27(3)	C1	N2	1.30(3)
C13	C14	1.40(3)			

[1]1-X,+Y,3/2-Z

Table S9. Bond Angles for **2H-Car-Ph**.

Atom	*Atom*	*Atom*	*Angle/°*	*Atom*	*Atom*	*Atom*	*Angle/°*
C3	C2	C25	114(2)	C13	C14	C15	127(2)
C1	C2	C3	120.6(14)	C16	C15	C14	122(3)
C1	C2	C25	125.3(16)	C15	C16	C17	112(3)
C2	C3	C4	120.5(18)	C16	C17	C18	121(2)
C3	C4	C19	121(2)	C17	C18	C13	131(2)
C5	C4	C3	118.2(17)	C20	C19	C4	122.7(15)
C5	C4	C19	120.7(15)	C23	C19	C4	123(2)
C4	C5	C6	121.7(15)	C23	C19	C20	114.4(19)
C5	C6	C7	133.5(14)	C19	C20	C24	126.5(19)
C5	C6	C1	120.2(19)	C22	C21	C24	121(2)
C1	C6	C7	106.2(17)	C21	C22	C23	123(2)
C8	C7	C6	108(2)	C22	C23	C19	120(2)
C11	C7	C6	132.6(17)	C21	C24	C20	114(2)
C11	C7	C8	119(3)	C26	C25	C2	122.0(18)
C7	C8	N2	104(2)	C26	C25	N1	123.5(19)
C9	C8	C7	124(2)	N1	C25	C2	114(2)
C9	C8	N2	132.0(17)	C27	C26	C25	123(2)
C8	C9	C10	115.8(19)	C26	C27	C28	114(3)
C8	C9	C29[1]	127(2)	C29	C28	C27	122.3(19)
C10	C9	C29[1]	117(3)	C28	C29	C9[1]	120(2)
C9	C10	C12	129(3)	N1	C29	C9[1]	120(3)
C7	C11	C12	124.0(18)	N1	C29	C28	119.5(19)
C10	C12	C11	108(2)	C29	N1	C25	117(2)
C13	C12	C10	134(3)	C2	C1	C6	118.3(17)
C13	C12	C11	117.4(19)	N2	C1	C2	131.3(14)
C12	C13	C14	119(2)	N2	C1	C6	110(2)
C12	C13	C18	136(3)	C1	N2	C8	111.8(14)
C18	C13	C14	103(2)				

[1]1-X,+Y,3/2-Z

Table S10. Hydrogen Atom Coordinates (Å×10^4) and Isotropic Displacement Parameters (Å^2×10^3) for **2H-Car-Ph**.

Atom	*x*	*y*	*z*	*U(eq)*
H3	6599.91	7319.54	8506.83	106
H5	6728.7	3997.39	7417.44	109
H1	4892.23	13960.08	7280.5	148
H10	5091.28	7031.11	5930.27	105
H11	6262.27	4276.57	6586.74	100
H14	5286.06	5766.04	5396.19	139
H15	5501.39	5708.11	4796.78	150
H16	5991.86	1822.59	4747.03	147
H17	6480.29	827.66	5408.67	140
H18	6274.67	1392.35	5992.1	131
H20	7080.46	2933.9	8754.49	138
H21	8271.91	2226.91	8760.61	142
H22	8081.25	3794.35	8075.73	140
H23	7409.13	5109.86	7718.09	135
H24	7769.96	1620.43	9125.28	145
H26	6301.61	11210.82	8713.5	109
H27	5951.51	13153.56	9156.67	112
H28	5248.03	11308.4	9036.96	108
H2	5331.25	8676.73	7418.11	96

Cryo-electron diffraction

As shown below, the weak data could be indexed and integrated in DIALS using a primitive monoclinic unit cell. There are 3 nonequivalent macrocycles per asymmetric unit (**Figure S30**). The structure is comparable to the water containing **2H-Car-Ph** structure with a triple unit cell along the b axis, which could be ascribed to a phase transition at low temperature. This tripling effect is not observed in the SAED patterns in **Figure S16**.

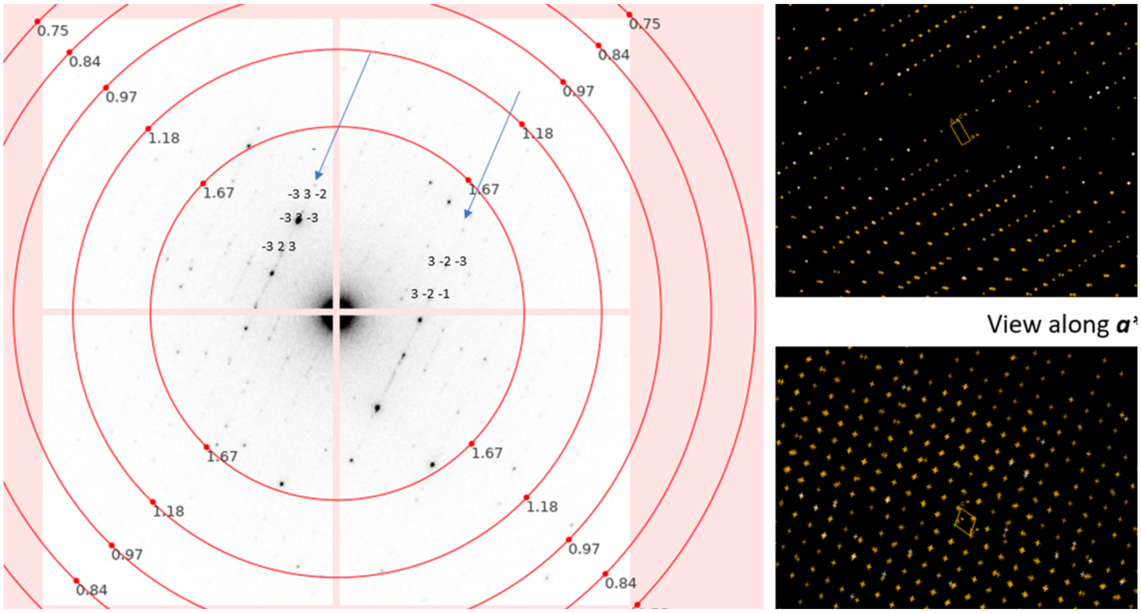

Figure S29. View of a typical image, showing the limited resolution (resolution rings depicted in red). Diffuse scattering is present (especially visible on the row indicated by the blue arrows). On the right, a view of the reciprocal space (based on the peaks caught by the peak hunting algorithm) is shown along two directions. The reciprocal unit cell is highlighted.

The data were cut at 1 Å resolution, as very few spots were visible on the images beyond this resolution. However, a plot of I/σ against the resolution indicates that the resolution of the data is closer to 1.5 Å.

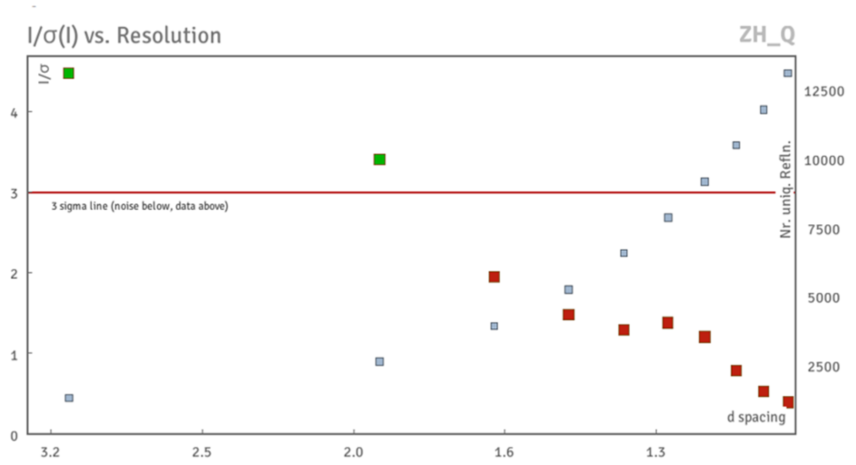

Figure S30. Plot of I/σ against resolution (large squares) for the merged data. The small squares indicate the number of unique reflections.

Due to the weakness of the data, only isotropic refinement was attempted. Restraints and constraints were used on the displacement parameters.

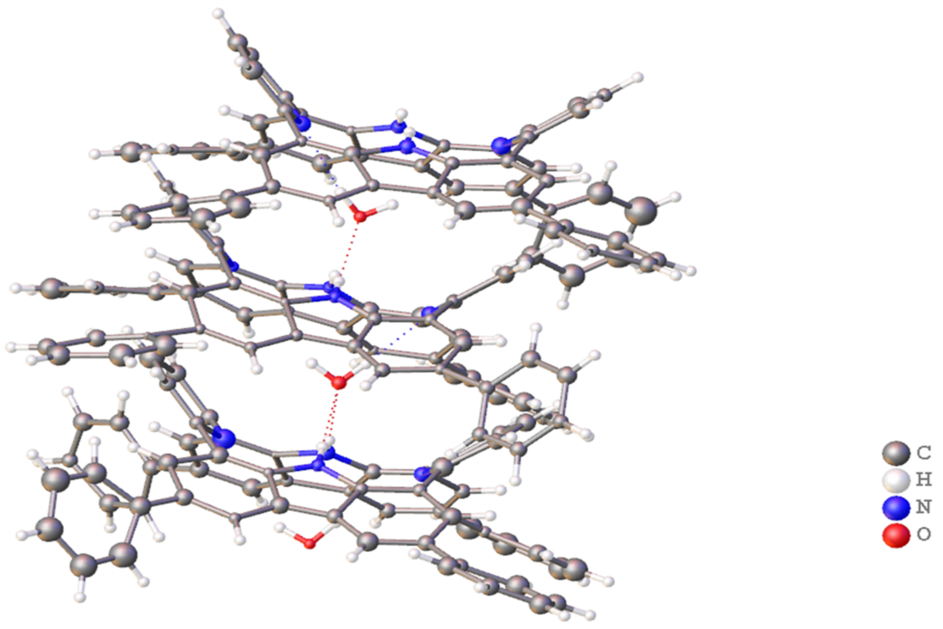

Figure S31. View of the asymmetric unit of the **2H-Car-Ph** structure formed from regular toluene at low temperature.

Table S11. Cryo-ED (100 K) and structure refinement for **2H-Car-Ph** nanosheets formed from regular toluene.

CCDC No.	2389928
Empirical formula	$C_{58}H_{38}N_4O$
Formula weight	806.97
Temperature/K	100
Crystal system	monoclinic
Space group	$P2_1/c$
a/Å	32.090(10)
b/Å	13.530(10)
c/Å	32.640(10)
α/°	90
β/°	105.46(5)
γ/°	90
Volume/Å^3	13659(12)
Z	12
ρ_{calc}g/cm^3	1.177
μ/mm^{-1}	0.000
F(000)	2117.0
Radiation	electron (λ = 0.0285)
2Θ range for data collection/°	0.052 to 1.612
Index ranges	$-31 \le h \le 31$, $-13 \le k \le 13$, $-25 \le l \le 25$
Reflections collected	46324
Independent reflections	8449 [R_{int} = 0.3819, R_{sigma} = 0.4459]
Data/restraints/parameters	8449/198/761
Goodness-of-fit on F^2	0.814
Final R indexes [I>=2σ (I)]	R_1 = 0.1399, wR_2 = 0.3070
Final R indexes [all data]	R_1 = 0.2821, wR_2 = 0.3585
Largest diff. peak/hole / e Å^{-3}	0.16/-0.16

Water molecules are in good positions for hydrogen bonding. For some hydrogen atoms, remaining electronic density was visible (**Figure S31**). However, the quality of the data makes the position of the hydrogen atoms unreliable.

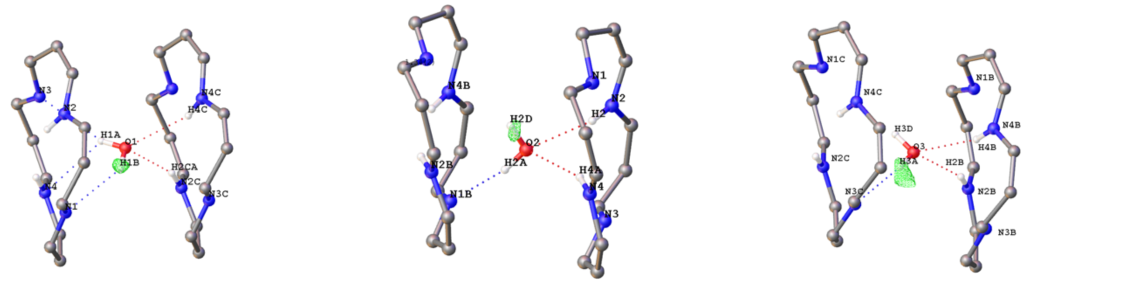

Donor	H	Acceptor	H...A (Å)	D...A (Å)	D-H....A (°)
O1	H1A	N3	2.50	3.32	160
O1	H1A	N4	2.5	3.05	123
O1	H1B	N1	2.77	3.52	150
N4C	H4C	O1	2.27	3.02	144
N2C	H2CA	O1	2.09	2.9	156

Donor	H	Acceptor	H...A (Å)	D...A (Å)	D-H....A (°)
O2	H2A	N1B	2.08	2.89	160
N2	H2	O2	2.17	3.00	161
N4	H4A	O2	2.01	2.79	151

Donor	H	Acceptor	H...A (Å)	D...A (Å)	D-H....A (°)
O3	H3A	N3C	2.09	2.92	165
N2B	H2B	O3	2.21	3.00	153
N4B	H4B	O3	2.09	2.86	149

Figure S32. View of the hydrogen atoms possibly involved in hydrogen bonds. In the table, unusual angles/distances are highlighted in violet. An omit map (difference Fourier map with the occupancies of the hydrogen atoms of the water molecules set to 0) is shown, revealing some electronic density at the position of some hydrogen atoms.

<u>Remarks on the quality of this dataset</u>

Electron diffraction data suffer from an inherent difficulty to meet the standard of the checkcif. They can have quite low completeness, due to the limited range of the tilt axis in the electron microscope. The intensities also suffer from multiple scattering and, when refining the structure in the kinematical approximation as was done for this dataset, the geometrical parameters (bond length, bond angles) are of lower quality. However, microED is known to provide reliable structures concerning the packing of the structure and the general connectivity, on very small crystals impossible to study with single-crystal X-ray diffraction, which is why we had to use it in our case.

There is an inherent limit to the quality that is obtainable for these types of materials due to the weak diffraction of the crystal at high angle and radiation damage occurring quite quickly – typical hallmarks of “soft matter”. In this particular case, the crystals tended to pack in the same orientation, making it difficult to reach a good completeness, even when combining many datasets from several crystals. The structure solved easily and the remaining density at some of the hydrogen positions confirmed that the quality of the data was sufficient to ensure the viability of the structure solution.

Table S12. Summary of lattice parameters obtained for **2H-Car-Ph** species from SAED, micro-electron diffraction and X-ray diffraction.

	***a* / Å**	***b* / Å**	***c* / Å**	***α* / °**	***β* / °**	***γ* / °**
**SAED (wet tol.)**	-	4.4	16.2	-	-	-
**SAED (reg. tol.)**	-	4.2	16.4	-	-	-
**SAED (dry tol.)**	17.2	7.6	-	-	-	-
**X-ray**	17.5299(3)	7.61020(10)	31.7286(4)	90	94.6650(10)	90
***μ*-ED (wet. tol., 273 K)**	33.2(5)	4.22(6)	33.0(5)	90	106.53(11)	90
**cryo-ED (reg. tol., 100 K)**	32.090(10)	13.530(10)	32.640(10)	90	105.45(5)	90
**cryo-ED (dry, glass, 100 K)**	17.638(10)	26.952(10)	18.059(10)	90	96.38(5)	90
**cryo-ED (dry tol., 100 K)**	17.061(3)	7.582(2)	30.736(6)	90	94.49(3)	90

Voids and pores

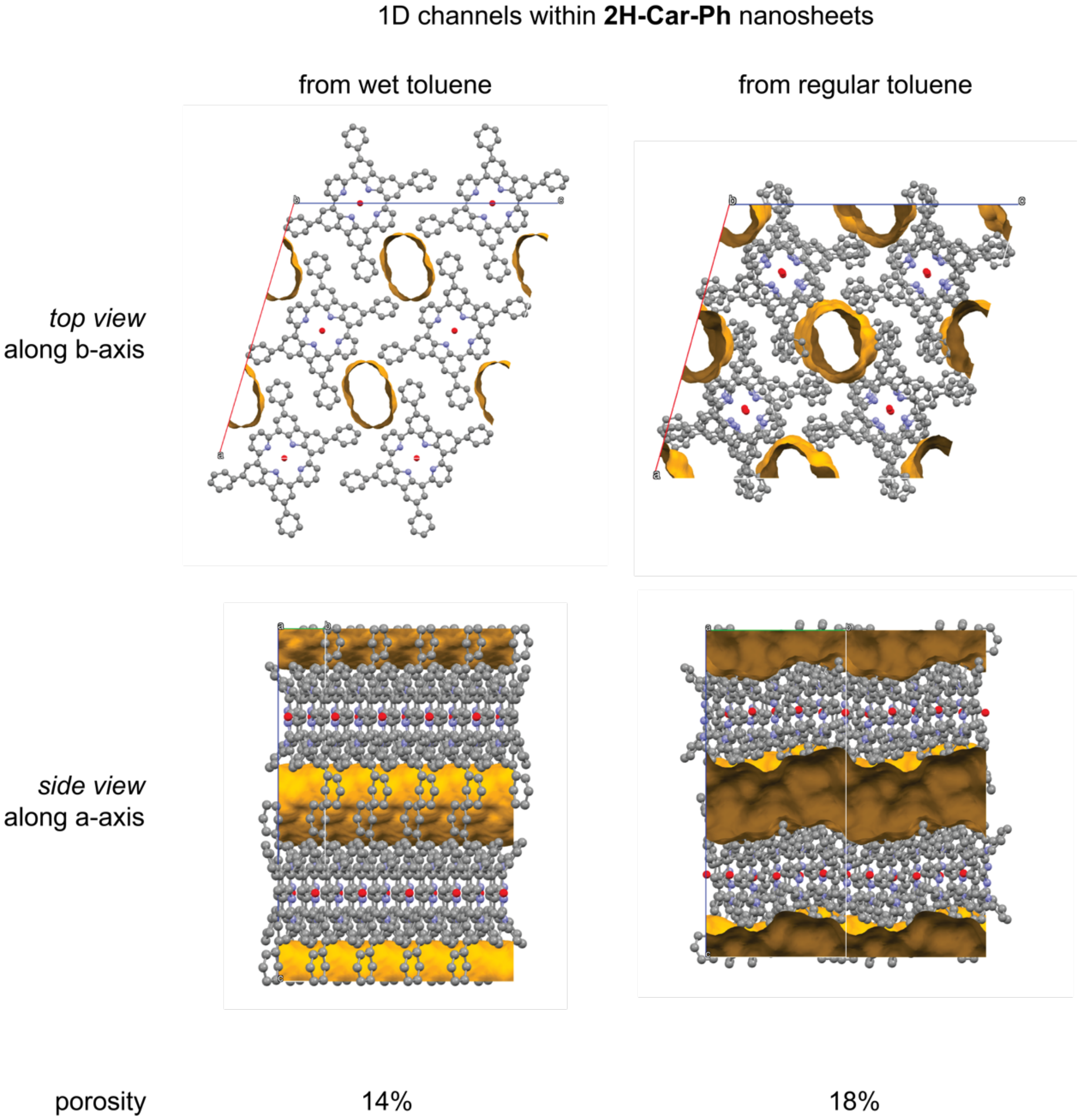

Figure S33. Nanosheets of **2H-Car-Ph** display porous 1D channels within their structure when dropcasted from wet or regular toluene. In both cases, the pores are located in the area shared between phenyl rings of four carpyridine units (*top view*) with the channels running parallel to columns of carpyridines (*side view*). These channels are more uniform when samples are dropcasted from wet toluene but becomes more porous when samples are dropcasted from regular toluene.

Table S13. Results of pore analysis of 2H-Car-Ph nanosheets when dropcasted from wet toluene (default settings).[30]

System Volume / Å^3	4432.35
System Mass / g·mol^{-1}	3227.788
System Density / g·cm^{-3}	1.209
Total surface area / Å^2	138.98
Total surface area per volume / m^2·cm^{-3}	313.56
Total surface area per mass / m^2·g^{-1}	259.3
Network-accessible surface area / Å^2	138.98
Network-accessible surface area per volume / m^2·cm^{-3}	313.56
Network-accessible surface area per mass / m^2·g^{-1}	259.3
Total helium volume / Å^3	733.045
Total helium volume per mass / cm^3·g^{-1}	0.137
Total geometric volume / Å^3	1544.401
Total geometric volume per mass / cm^3·g^{-1}	0.288
Network-accessible helium volume / Å^3	733.045
Network-accessible helium volume / cm^3·g^{-1}	0.137
Network-accessible geometric volume / Å^3	1542.165
Network-accessible geometric volume / cm^3·g^{-1}	0.288
Pore limiting diameter / Å	4.33
Maximum pore diameter / Å	4.90
Number of percolated dimensions	1

Table S14. Results of pore analysis of 2H-Car-Ph nanosheets when dropcasted from regular toluene (default settings).[30]

System Volume / $Å^3$	13088.325
System Mass / $g \cdot mol^{-1}$	9630.953
System Density / $g \cdot cm^{-3}$	1.222
Total surface area / $Å^2$	440.18
Total surface area per volume / $m^2 \cdot cm^{-3}$	336.32
Total surface area per mass / $m^2 \cdot g^{-1}$	275.24
Network-accessible surface area / $Å^2$	440.18
Network-accessible surface area per volume / $m^2 \cdot cm^{-3}$	336.32
Network-accessible surface area per mass / $m^2 \cdot g^{-1}$	275.24
Total helium volume / $Å^3$	2747.774
Total helium volume per mass / $cm^3 \cdot g^{-1}$	0.172
Total geometric volume / $Å^3$	4561.703
Total geometric volume per mass / $cm^3 \cdot g^{-1}$	0.285
Network-accessible helium volume / $Å^3$	2747.615
Network-accessible helium volume / $cm^3 \cdot g^{-1}$	0.172
Network-accessible geometric volume / $Å^3$	4858.502
Network-accessible geometric volume / $cm^3 \cdot g^{-1}$	0.285
Pore limiting diameter / Å	4.51
Maximum pore diameter / Å	4.85
Number of percolated dimensions	1

Powder X-ray diffraction

Vapor annealing interconversion experiments

2D diffraction data were collected on a Rigaku XtaLAB Synergy, Dualflex, HyPix-Arc 150° diffractometer using Cu Kα radiation (λ = 1.54184 Å). The samples were scratched and grounded between two microscope slides. The powder was deposited on a Hampton cryoloop. The program suite *CrysAlisPro*[10] was used to convert them into conventional 1D powder diffraction diagrams. Vapor annealing experiments were conducted to investigate possible interconversion between the dry and wet phases. The dry and wet samples were prepared by dropcasting 1 mM solutions of **2H-Car-Ph** onto glass slides. To verify whether the 2D nanosheets obtained were identical to those observed on Cu/C TEM grids, we performed X-ray powder diffraction (PXRD) experiments and compared the experimental PXRD patterns with simulated data.

The PXRD pattern of the wet sample matched well with the simulated data. Using PXRD, we observed the conversion of wet assemblies to dry assemblies through annealing in dry toluene. Vapor annealing the wet sample under dry toluene resulted in complete conversion to a new phase, which matched the simulated PXRD data of the dry assemblies. However, the subsequent reverse annealing under wet toluene vapor was unsuccessful; only the dry assemblies were detected based on PXRD data.

Interestingly, the dry 2D nanosheet prepared by dropcasting onto a glass slide exhibited a different phase when compared to the PXRD data of previously observed dry assemblies. This suggests that the surface plays a critical role in the self-assembly process of **2H-Car-Ph**.

To obtain more structural information about the dry phase formed on the glass slide, we transferred the 2D nanosheets from the glass slide to a Cu/C grid for cryo-electron diffraction (cryo-ED, 100 K) experiments. The obtained unit cell parameters (Fig. S36) differed from those of the dry assemblies formed directly on the Cu/C grid. However, the quality of the data was insufficient to allow for structural refinement.

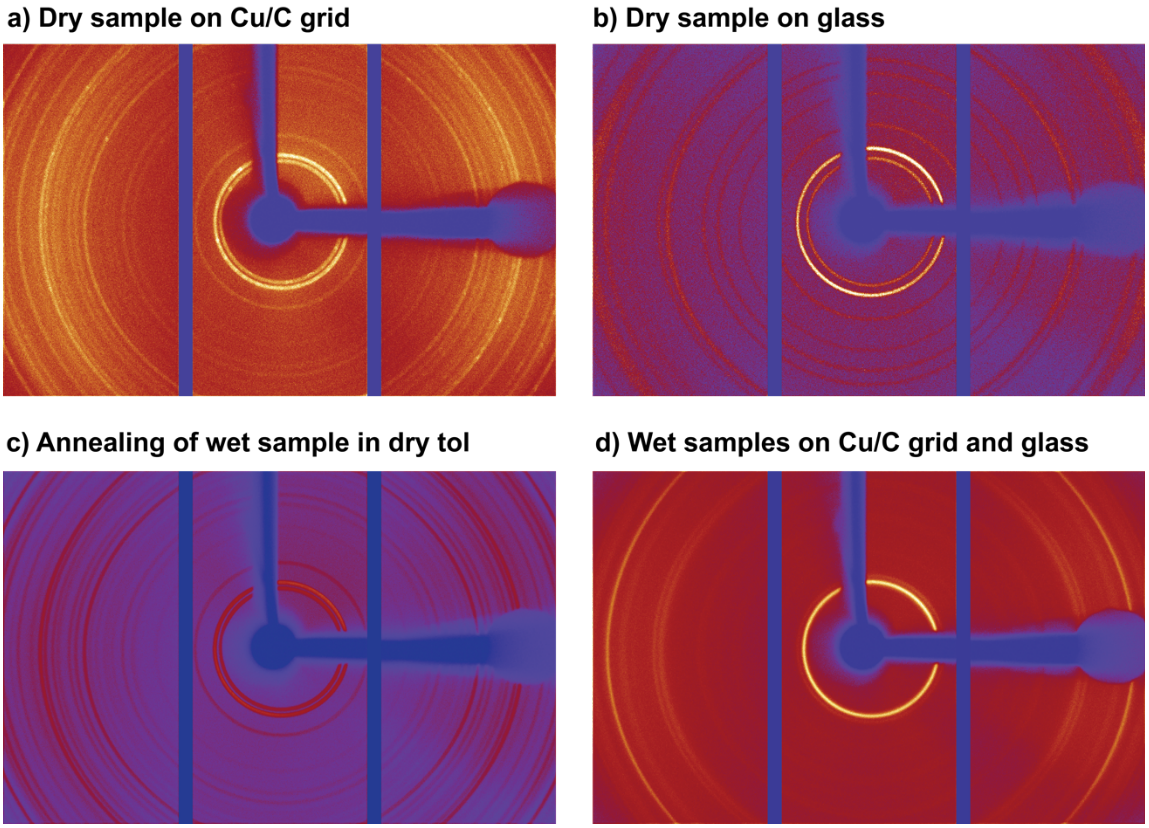

Figure S34. 2D Powder X-ray diffraction patterns of a) dry **2H-Car-Ph** nanosheets dropcasted on a Cu/C TEM grid, b) dry **2H-Car-Ph** nanosheets dropcasted on a glass slide, c) **2H-Car-Ph** nanosheets dropcasted from wet toluene onto a glass slide and annealed in dry toluene and d) **2H-Car-Ph** nanosheets dropcasted from wet toluene onto a Cu/C TEM grid and a glass slide.

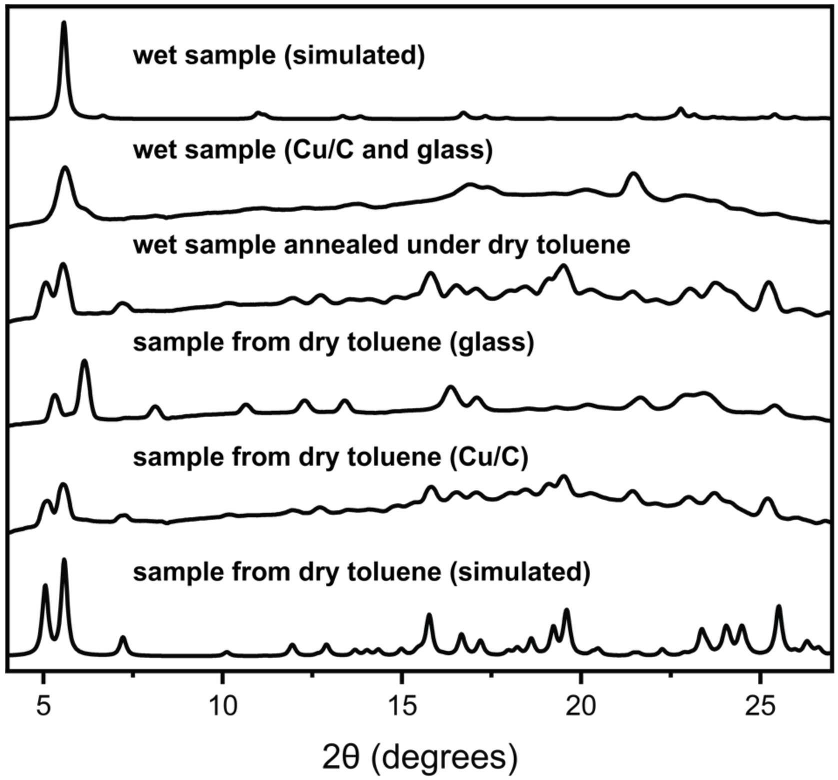

Figure S35. 1D plots of powder X-ray diffraction patterns of **2H-Car-Ph** nanosheets under different conditions. Two phases are observed for the nanosheets formed from dry toluene, depending upon the surface. Annealing of the wet sample under dry toluene affords the same phase as observed for dry nanosheets on Cu/C TEM grids.

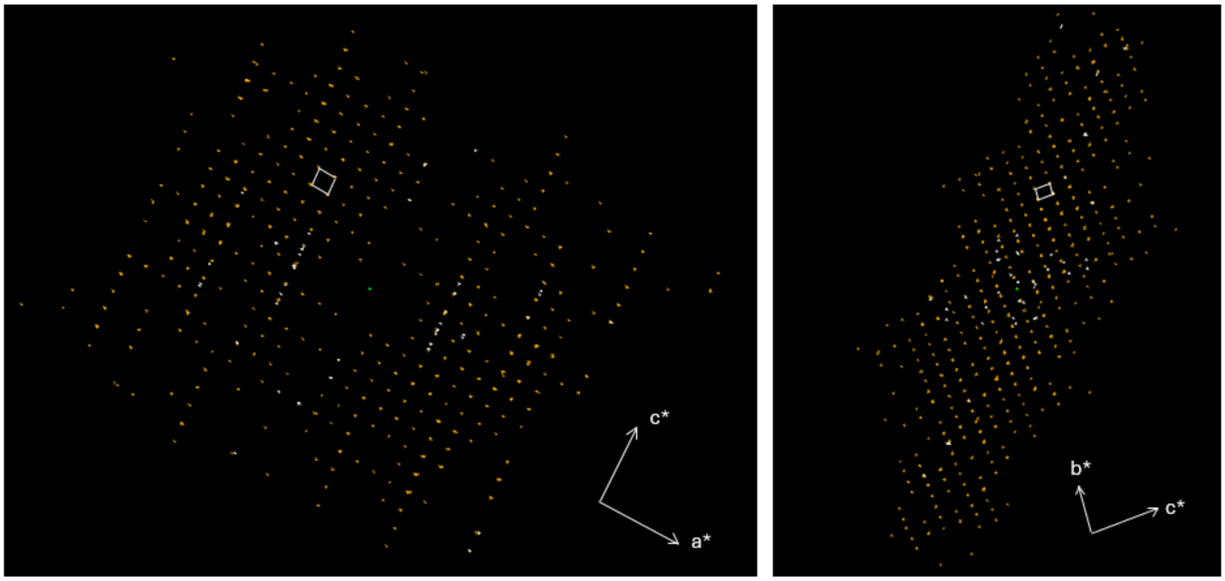

Figure S36. Views of the reciprocal space of dry **2H-Car-Ph** nanosheets formed on a glass surface (based on the peaks caught by the peak hunting algorithm) shown along *b** (left) and *a** (right). The reciprocal unit cell (monoclinic with a = 17.1 Å, b = 27.3 Å, c = 18.1 Å and β = 94.7°) is highlighted.

Dynamic Light Scattering

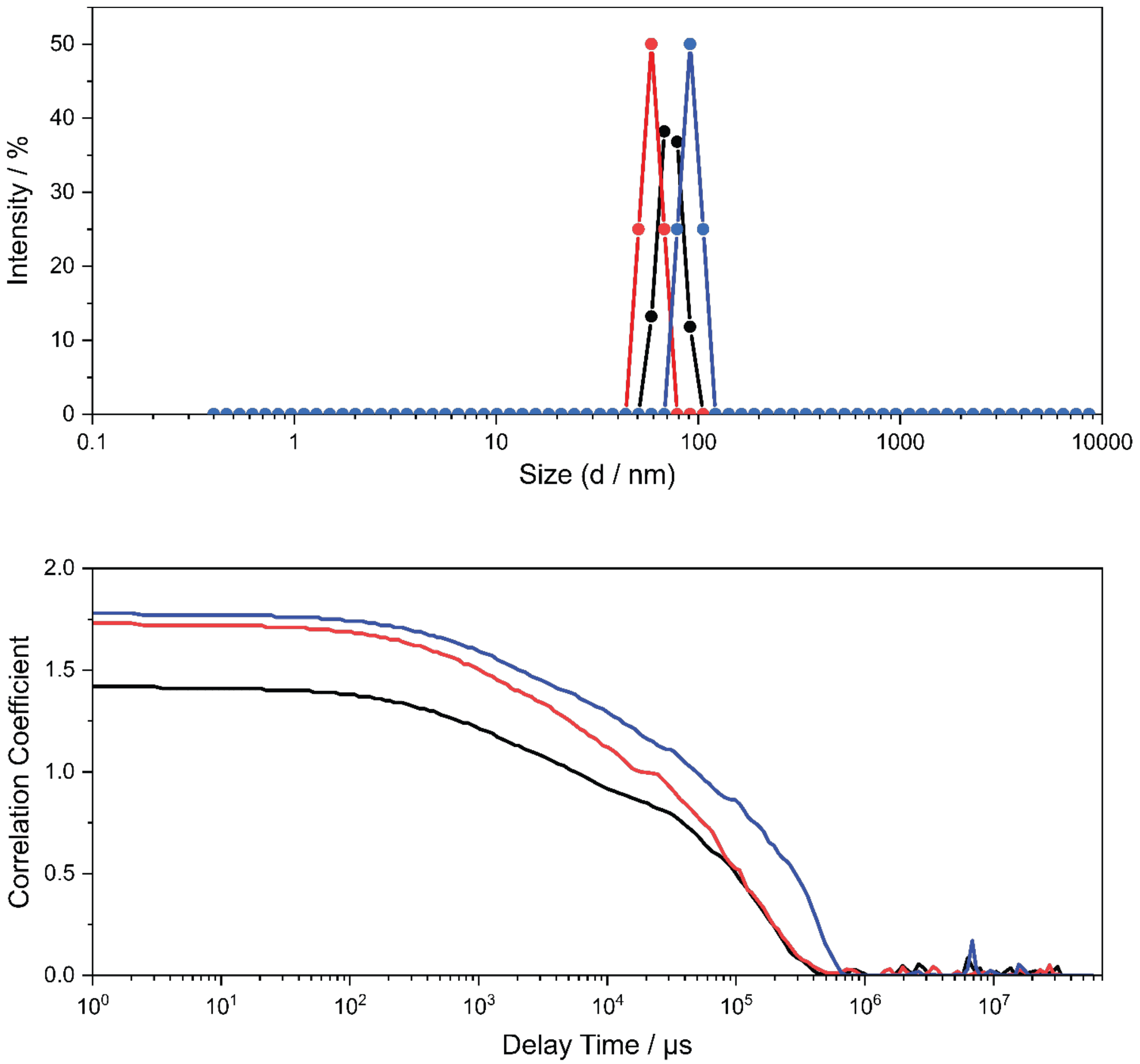

Figure S37. Dynamic light scattering size distribution, top, of a 600 µM solution of **2H-Car-Ph** in dry toluene at 25 °C showing the presence of aggregates (100 nm) and its correlation coefficient plot, bottom.

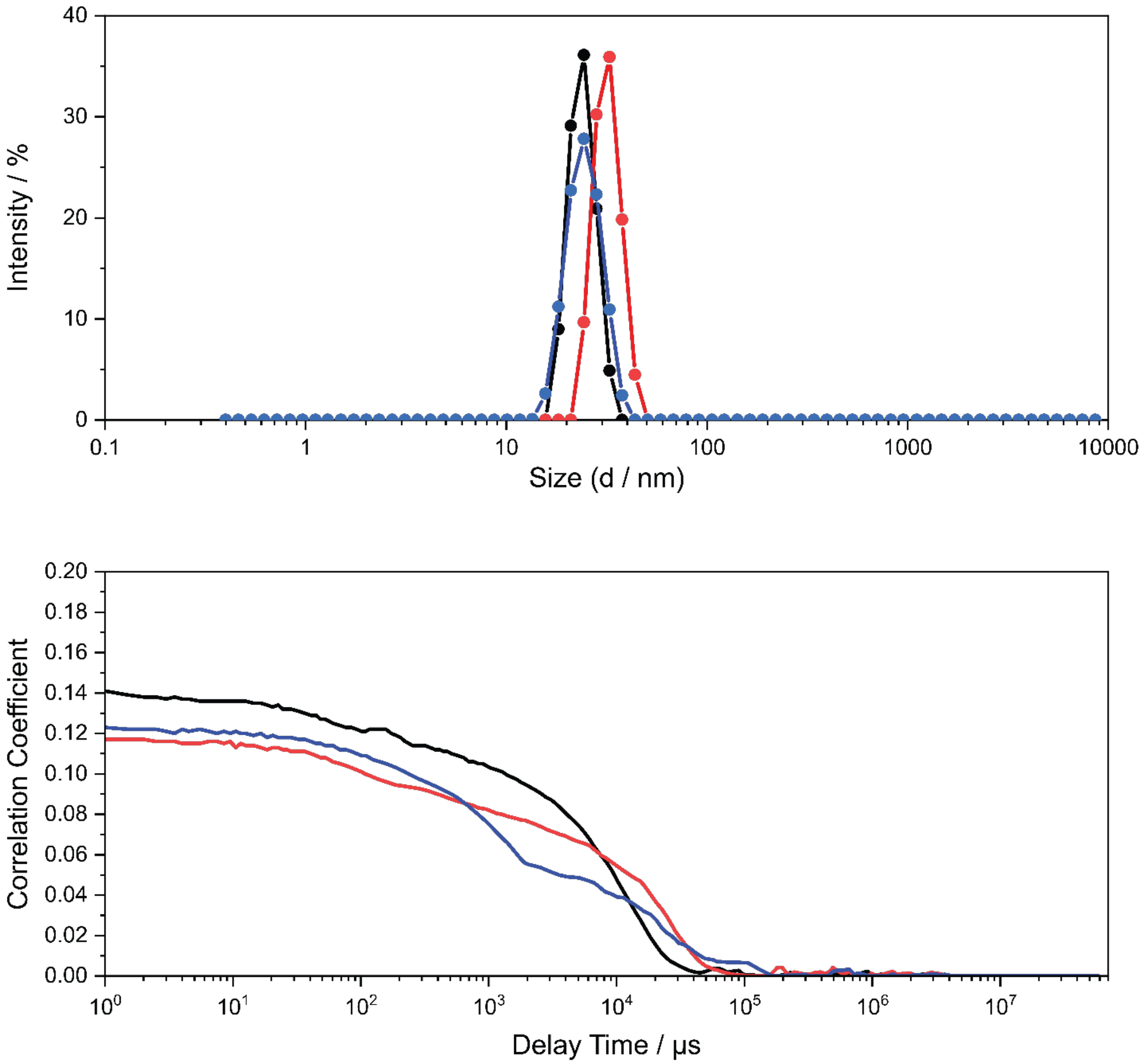

Figure S38. Dynamic light scattering size distribution, top, of a 600 µM solution of **2H-Car-Ph** in dry toluene at 70 °C showing the presence of smaller aggregates (30 nm) and its correlation coefficient plot, bottom.

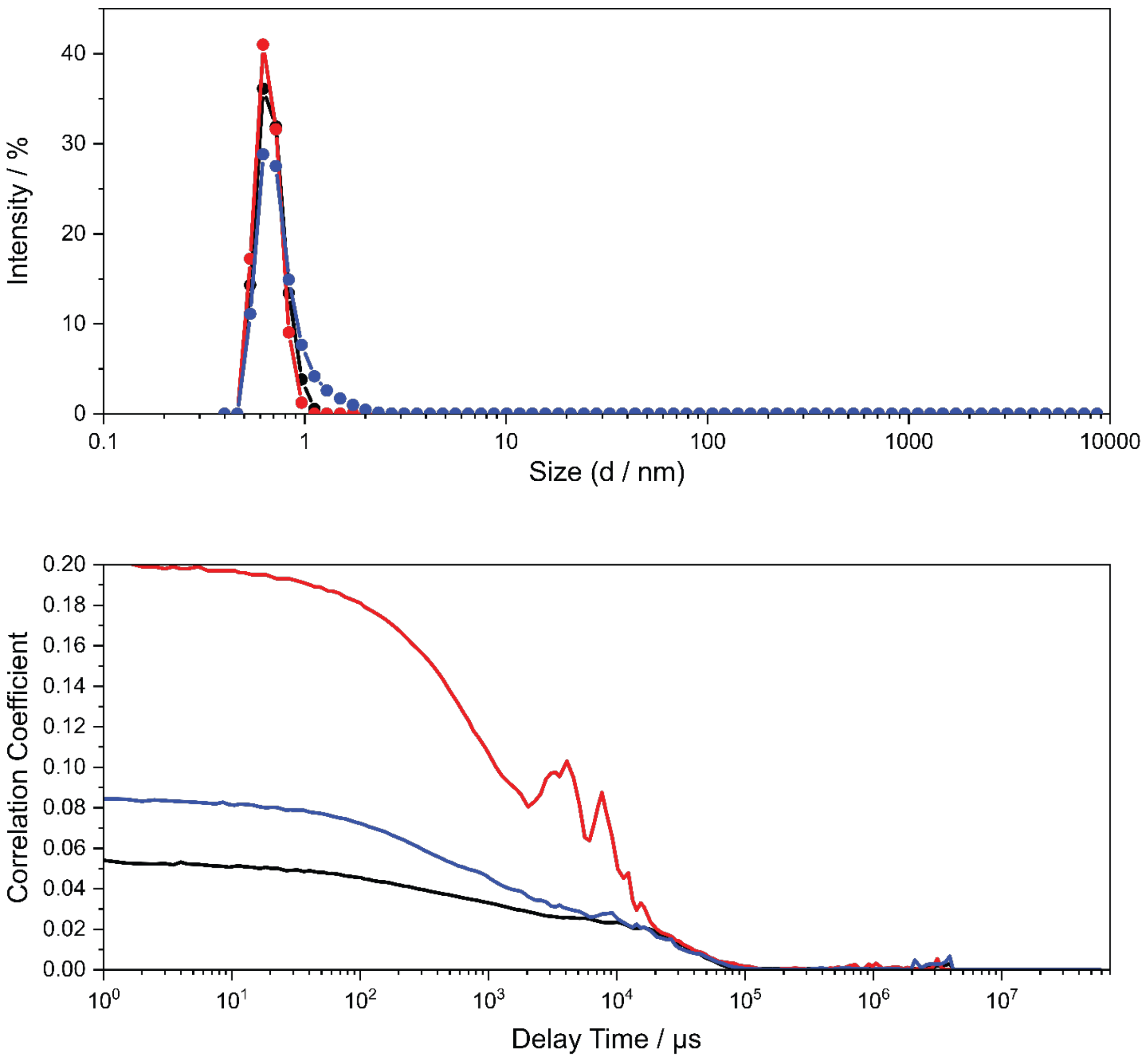

Figure S39. Dynamic light scattering size distribution, top, of a 600 μM solution of **2H-Car-Ph** in dry toluene at 90 °C showing the presence of monomers and its correlation coefficient plot, bottom.

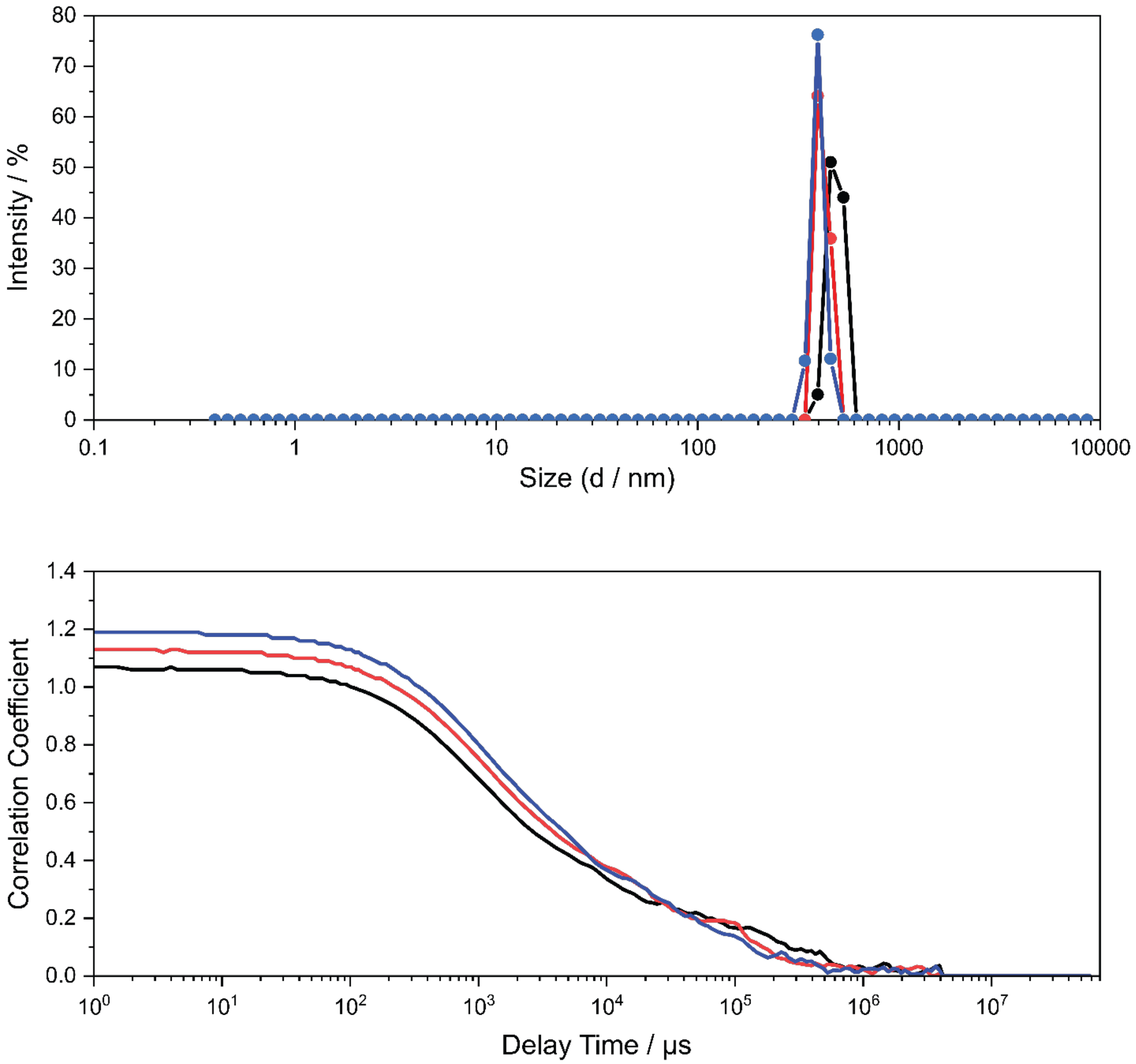

Figure S40. Dynamic light scattering size distribution, top, of a 600 μM solution of **2H-Car-Ph** in dry toluene cooled to 25 °C after heating to 90 °C showing the presence of aggregates (500 nm) and its correlation coefficient plot, bottom.

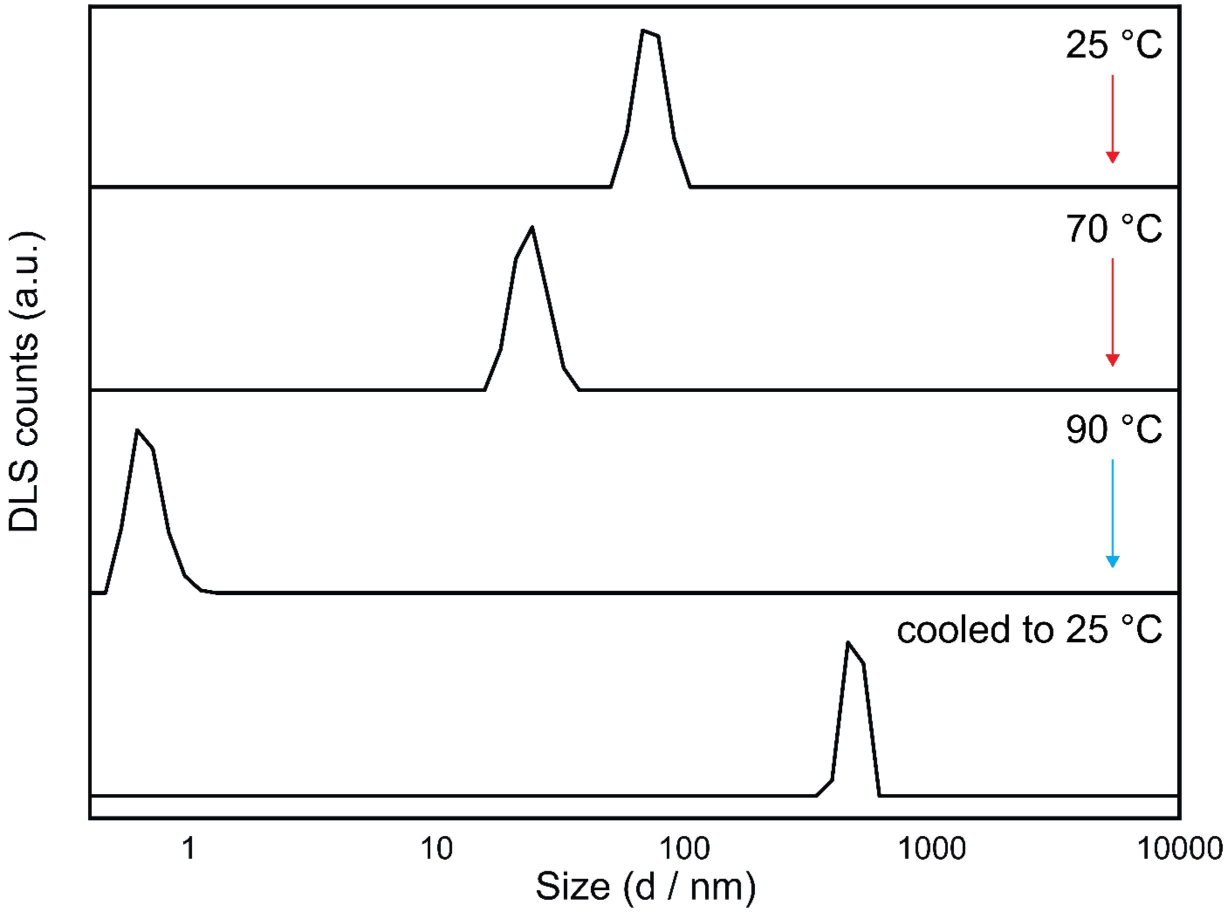

Figure S41. Comparison of dynamic light scattering plots of a 600 µM solution of **2H-Car-Ph** in dry toluene at different consecutive temperatures. The final trace has been acquired after cooling for 1d. Aggregates are dissolved upon heating and are then reformed after cooling back to 25 °C.

Supplementary References

(1) Frisch, M. J.; Trucks, G. W.; Schlegel, H. B.; Scuseria, G. E.; Robb, M. a.; Cheeseman, J. R.; Scalmani, G.; Barone, V.; Petersson, G. a.; Nakatsuji, H.; Li, X.; Caricato, M.; Marenich, a. V.; Bloino, J.; Janesko, B. G.; Gomperts, R.; Mennucci, B.; Hratchian, H. P.; Ortiz, J. V.; Izmaylov, a. F.; Sonnenberg, J. L.; Williams; Ding, F.; Lipparini, F.; Egidi, F.; Goings, J.; Peng, B.; Petrone, A.; Henderson, T.; Ranasinghe, D.; Zakrzewski, V. G.; Gao, J.; Rega, N.; Zheng, G.; Liang, W.; Hada, M.; Ehara, M.; Toyota, K.; Fukuda, R.; Hasegawa, J.; Ishida, M.; Nakajima, T.; Honda, Y.; Kitao, O.; Nakai, H.; Vreven, T.; Throssell, K.; Montgomery Jr., J. a.; Peralta, J. E.; Ogliaro, F.; Bearpark, M. J.; Heyd, J. J.; Brothers, E. N.; Kudin, K. N.; Staroverov, V. N.; Keith, T. a.; Kobayashi, R.; Normand, J.; Raghavachari, K.; Rendell, a. P.; Burant, J. C.; Iyengar, S. S.; Tomasi, J.; Cossi, M.; Millam, J. M.; Klene, M.; Adamo, C.; Cammi, R.; Ochterski, J. W.; Martin, R. L.; Morokuma, K.; Farkas, O.; Foresman, J. B.; Fox, D. J. Gaussian 16, Revision C.01. 2016, p Gaussian 16, Revision C.01, Gaussian, Inc., Wallin.

(2) Vosko, S. H.; Wilk, L.; Nusair, M. Accurate Spin-Dependent Electron Liquid Correlation Energies for Local Spin Density Calculations: A Critical Analysis. *Can. J. Phys.* **1980**, *58* (8), 1200–1211. https://doi.org/10.1139/p80-159.

(3) Lee, C.; Yang, W.; Parr, R. G. Development of the Colle-Salvetti Correlation-Energy Formula into a Functional of the Electron Density. *Phys. Rev. B* **1988**, *37* (2), 785–789. https://doi.org/10.1103/PhysRevB.37.785.

(4) Becke, A. D. Density-Functional Thermochemistry. III. The Role of Exact Exchange. *J. Chem. Phys.* **1993**, *98* (7), 5648–5652. https://doi.org/10.1063/1.464913.

(5) Stephens, P. J.; Devlin, F. J.; Chabalowski, C. F.; Frisch, M. J. Ab Initio Calculation of Vibrational Absorption and Circular Dichroism Spectra Using Density Functional Force Fields. *J. Phys. Chem.* **1994**, *98* (45), 11623–11627. https://doi.org/10.1021/j100096a001.

(6) Rassolov, V. A.; Ratner, M. A.; Pople, J. A.; Redfern, P. C.; Curtiss, L. A. 6-31G* Basis Set for Third-row Atoms. *J. Comput. Chem.* **2001**, *22* (9), 976–984. https://doi.org/10.1002/jcc.1058.

(7) Grimme, S.; Antony, J.; Ehrlich, S.; Krieg, H. A Consistent and Accurate Ab Initio Parametrization of Density Functional Dispersion Correction (DFT-D) for the 94 Elements H-Pu. *J. Chem. Phys.* **2010**, *132* (15), 154104. https://doi.org/10.1063/1.3382344.

(8) Grimme, S.; Ehrlich, S.; Goerigk, L. Effect of the Damping Function in Dispersion Corrected Density Functional Theory. *J. Comput. Chem.* **2011**, *32* (7), 1456–1465. https://doi.org/10.1002/jcc.21759.

(9) Marenich, A. V.; Cramer, C. J.; Truhlar, D. G. Universal Solvation Model Based on Solute Electron Density and on a Continuum Model of the Solvent Defined by the Bulk Dielectric Constant and Atomic Surface Tensions. *J. Phys. Chem. B* **2009**, *113* (18), 6378–6396. https://doi.org/10.1021/jp810292n.

(10) Rigaku Corporation. Rigaku Oxford Diffraction, CrysAlisPro Software System. Wroclaw, Poland 2021.

(11) Coppens, P.; Leiserowitz, L.; Rabinovich, D. Calculation of Absorption Corrections for Camera and Diffractometer Data. *Acta Crystallogr.* **1965**, *18* (6), 1035–1038. https://doi.org/10.1107/S0365110X65002487.

(12) Sheldrick, G. M. SHELXT – Integrated Space-Group and Crystal-Structure Determination. *Acta Crystallogr. A Found. Adv* **2015**, *71* (1), 3–8. https://doi.org/10.1107/S2053273314026370.

(13) Maslen, E. N., Fox, A. G., O'Keefe, M. A. *International Tables for Crystallography*; Wilson, A. J. C., Ed.; Kluwer Academic Publishers, 2004.
(14) Stewart, R.F., Davidson, E. R., S. W. T. Coherent X-Ray Scattering for the Hydrogen Atom in the Hydrogen Molecule. *J. Chem. Phys.* **1965**, *42*, 3175–3187.
(15) Bruker AXS Inc. APEX4. Apex Suite of Crystallographic Software: Madison WI USA 2022.
(16) Bruker AXS Inc. SAINT v8.30A. Madison WI USA 2012.
(17) Bruker AXS Inc. SADABS V2016/2. Madison WI USA 2016.
(18) Sheldrick, G. M. SHELXT - Integrated Space-Group and Crystal-Structure Determination. *Acta Crystallogr. A* **2015**, *71* (1), 3–8. https://doi.org/10.1107/S2053273314026370.
(19) Sheldrick, G. M. A Short History of SHELX. *Acta Crystallogr. A* **2008**, *64* (1), 112–122. https://doi.org/10.1107/S0108767307043930.
(20) Hübschle, C. B.; Sheldrick, G. M.; Dittrich, B. *ShelXle*: A Qt Graphical User Interface for *SHELXL*. *J. Appl. Crystallogr.* **2011**, *44* (6), 1281–1284. https://doi.org/10.1107/S0021889811043202.
(21) Winter, G.; Beilsten-Edmands, J.; Devenish, N.; Gerstel, M.; Gildea, R. J.; McDonagh, D.; Pascal, E.; Waterman, D. G.; Williams, B. H.; Evans, G. DIALS as a Toolkit. *Prot. Sci.* **2022**, *31* (1), 232–250. https://doi.org/10.1002/pro.4224.
(22) Sheldrick, G. M. A Short History of *SHELX*. *Acta Crystallogr. A* **2008**, *64* (1), 112–122. https://doi.org/10.1107/S0108767307043930.
(23) Zhang, Y.-L.; Ran, Q.; Wang, Q.; Tian, Q.-S.; Kong, F.-C.; Fan, J.; Liao, L.-S. High-Performance Sky-Blue Phosphorescent Organic Light-Emitting Diodes Employing Wide-Bandgap Bipolar Host Materials with Thermally Activated Delayed Fluorescence Characteristics. *Org. Electron.* **2020**, *81*, 105660. https://doi.org/10.1016/j.orgel.2020.105660.
(24) Rembiak, A.; Koskinen, A. Versatile Synthesis of Symmetrical Carbazole-Based Ligand Precursors via Regioselective Aromatic Bromination. *Synthesis* **2015**, *47* (21), 3347–3353. https://doi.org/10.1055/s-0034-1378825.
(25) Clark, R. C.; Reid, J. S. The Analytical Calculation of Absorption in Multifaceted Crystals. *Acta Crystallogr. A* **1995**, *51* (6), 887–897. https://doi.org/10.1107/S0108767395007367.
(26) Dolomanov, O. V.; Bourhis, L. J.; Gildea, R. J.; Howard, J. A. K.; Puschmann, H. OLEX2 : A Complete Structure Solution, Refinement and Analysis Program. *J. Appl. Crystallogr.* **2009**, *42* (2), 339–341. https://doi.org/10.1107/S0021889808042726.
(27) Sheldrick, G. M. SHELXT – Integrated Space-Group and Crystal-Structure Determination. *Acta Crystallogr. A Found. Adv* **2015**, *71* (1), 3–8. https://doi.org/10.1107/S2053273314026370.
(28) Sheldrick, G. M. Crystal Structure Refinement with SHELXL. *Acta Crystallogr. C Struct. Chem.* **2015**, *71* (1), 3–8. https://doi.org/10.1107/S2053229614024218.
(29) Spek, A. L. Structure Validation in Chemical Crystallography. *Acta Crystallogr. D Biol. Crystallogr.* **2009**, *65* (2), 148–155. https://doi.org/10.1107/S090744490804362X.
(30) Sarkisov, L.; Bueno-Perez, R.; Sutharson, M.; Fairen-Jimenez, D. Materials Informatics with PoreBlazer v4.0 and the CSD MOF Database. *Chem. Mater.* **2020**, *32* (23), 9849–9867. https://doi.org/10.1021/acs.chemmater.0c03575.
